# Transposon-induced epigenetic silencing in the X chromosome as a novel form of *dmrt1* expression regulation during sex determination in the fighting fish

**DOI:** 10.1186/s12915-021-01205-y

**Published:** 2022-01-07

**Authors:** Le Wang, Fei Sun, Zi Yi Wan, Zituo Yang, Yi Xuan Tay, May Lee, Baoqing Ye, Yanfei Wen, Zining Meng, Bin Fan, Yuzer Alfiko, Yubang Shen, Francesc Piferrer, Axel Meyer, Manfred Schartl, Gen Hua Yue

**Affiliations:** 1 Molecular Population Genetics & Breeding Group, Temasek Life Sciences Laboratory, Singapore, 117604 Singapore; 2 School of Life Sciences, Sun Yat-sen University, Guangzhou, 510275 China; 3 Department of Food and Environmental Engineering, Yangjiang Polytechnic, Yangjiang, 529500 China; 4 Biotech Lab, Wilmar International, Jakarta, Indonesia; 5 Key Laboratory of Exploration and Utilization of Aquatic Genetic Resources, Shanghai Ocean University, Shanghai, 201306 China; 6 Institute of Marine Sciences (ICM), Spanish National Research Council (CSIC), 08003 Barcelona, Spain; 7 Department of Biology, University of Konstanz, 78457 Konstanz, Germany; 8 Developmental Biochemistry, Biocenter, University of Wuerzburg, 97074 Wuerzburg, Germany; 9 The Xiphophorus Genetic Stock Center, Department of Chemistry and Biochemistry, Texas State University, San Marcos, TX 78666 USA; 10 Department of Biological Sciences, National University of Singapore, Singapore, 117543 Singapore; 11 School of Biological Sciences, Nanyang Technological University, Singapore, 637551 Singapore

**Keywords:** Teleost, Sex determination, *dmrt1*, Transposon, Epigenetic regulation, Sex reversal

## Abstract

**Background:**

Fishes are the one of the most diverse groups of animals with respect to their modes of sex determination, providing unique models for uncovering the evolutionary and molecular mechanisms underlying sex determination and reversal. Here, we have investigated how sex is determined in a species of both commercial and ecological importance, the Siamese fighting fish *Betta splendens*.

**Results:**

We conducted association mapping on four commercial and two wild populations of *B. splendens*. In three of the four commercial populations, the master sex determining (MSD) locus was found to be located in a region of ~ 80 kb on LG2 which harbours five protein coding genes, including *dmrt1*, a gene involved in male sex determination in different animal taxa. In these fish, *dmrt1* shows a male-biased gonadal expression from undifferentiated stages to adult organs and the knockout of this gene resulted in ovarian development in XY genotypes. Genome sequencing of XX and YY genotypes identified a transposon, *drbx1*, inserted into the fourth intron of the X-linked *dmrt1* allele. Methylation assays revealed that epigenetic changes induced by *drbx1* spread out to the promoter region of *dmrt1*. In addition, *drbx1* being inserted between two closely linked *cis*-regulatory elements reduced their enhancer activities. Thus, epigenetic changes, induced by *drbx1*, contribute to the reduced expression of the X-linked *dmrt1* allele, leading to female development. This represents a previously undescribed solution in animals relying on *dmrt1* function for sex determination. Differentiation between the X and Y chromosomes is limited to a small region of ~ 200 kb surrounding the MSD gene. Recombination suppression spread slightly out of the SD locus. However, this mechanism was not found in the fourth commercial stock we studied, or in the two wild populations analysed, suggesting that it originated recently during domestication.

**Conclusions:**

Taken together, our data provide novel insights into the role of epigenetic regulation of *dmrt1* in sex determination and turnover of SD systems and suggest that fighting fish are a suitable model to study the initial stages of sex chromosome evolution.

**Supplementary Information:**

The online version contains supplementary material available at 10.1186/s12915-021-01205-y.

## Background

The sex determination (SD) system, its interaction and coevolution with sex chromosomes are among the evolutionary innovations, which display a most spectacular plasticity [1]. Recent studies in teleosts have provided novel insights into how master sex determining (MSD) genes emerge and coevolve together with sex chromosomes, leading to the evolutionary path of autosomes to sex chromosomes [2–9]. Teleosts are a group of vertebrates with an amazing diversity of sexual systems (gonochorism, different forms of hermaphroditism, androdioecy and unisexuality) and an equally diverse array of SD mechanisms, from genetic to environmental SD. In fish, genetic SD can have a monogenic or polygenic basis, and can even be influenced by the interplay between genetic and environmental factors. Hence, sex reversal is very common even in gonochoristic teleosts [10–12]. SD systems can evolve independently following chromosome structural variation and allelic diversification and promote rapid turnover of sex chromosomes and hence of SD mechanisms [3, 5, 13]. Thus, SD systems can differ among closely related species and even among different populations within a species [14–16]. Such plasticity of SD systems is intriguing and has raised the interest of evolutionary biologists to understand the rewiring of gene regulatory networks to adapt to such rapid turnover of SD systems.

With the independent turnover of SD systems and a distinct evolutionary history, teleosts provide unique opportunities to investigate sex chromosome evolution at different stages of differentiation [3, 17]. Particularly in the case of origins of MSD genes from duplication of an autosomal gene, the non-homology between the proto-sex chromosomes initiates their differentiation [3]. According to the classical view of sex chromosome evolution, such sequence divergence further suppresses recombination, resulting in increased sequence divergence between the pair of sex chromosomes [18]. Genetic divergence accumulates with time. As a consequence, sex chromosomes eventually evolve to become morphologically distinct sex chromosomes and are sustained by balancing selection, if no additional SD system turnover occurs during this evolutionary process [19].

Teleosts also provide unique opportunities to study the functional transition of key players that may swap the sex of organisms. Compared to the other vertebrate groups, MSD genes in fish are extremely diverse. They are mainly derived from the *mab-3 related transcription factor 1* gene (*dmrt1*), members of the *soxa* and *b1* gene families and components of the TGF-β signalling pathway. MSD genes originate either by duplication followed by neo-functionalization or allelic diversification [10]. For example, duplicates of anti-Müllerian hormone (*amh*) or *dmrt1* translocated to a different chromosome and evolved to become MSD genes in Nile tilapia (*Oreochromis niloticus*) [20], Northern pike (*Esox lucius*) [3], and medaka (*Oryzias latipes*) [8, 9], respectively. For allelic diversification, the mechanisms of MSD genes are classified into two categories. One is that the associated alternative variations do not affect the expression patterns of MSD genes but alter the effectiveness of interactions with its downstream genes, e.g. a missense SNP in anti-Müllerian hormone receptor type II (*amhr2*) reduces AMH signalling and evolved as the MSD gene in pufferfish (*Takifugu rubripes*) [5]. The other one is variation that occurs in linked *cis*-regulatory elements of MSD genes, thus altering expression patterns, e.g. in the Luzon ricefish (*Oryzias luzonensis*), where Y-specific SNPs in the promoter region of *gonadal soma derived growth factor* (*gsdf*) increase its expression in males and drives this gene acting as an MSD gene [7].

The Siamese fighting fish, *Betta splendens*, naturally distributed in the Mekong River basin, is an important fish species in the ornamental fish industry [21]. This fish has been bred for exhibition contests since the mid-19th century in Thailand and become a popular ornamental fish world-wide in the last century [21]. The fighting fish reaches sexual maturity at the age of 3–4 months [21]. There are evidences for both sex chromosome systems, polygenic sex determination, and influence of environmental factors such as temperature [21, 22]. Therefore, this fish is a perfectly suited model to study sex determination and sex reversal, as well as sex chromosome evolution. However, little is known so far in this respect in the fighting fish.

Consequently, the objectives of this study were to examine the pattern of SD and the MSD gene, determine the state of differentiation between sex chromosomes and fluctuations of the SD system. By inheritance tests, genome scanning, whole genome sequencing, expression analyses, and gene knockout, we observed that *B. splendens* has a XY sex chromosome system in all but one commercial stock analysed. Environmental factors may also affect sexual differentiation as frequent sex reversal has been observed. The sex locus was located to a very narrow region on LG2, with recombination suppression slightly spreading out of this region. We found that differentiation between X and Y chromosomes is in an initial stage. We also detected that only one gene, from the differentiated segment, namely *dmrt1*, showed male biased expression before gonad differentiation. Knockout of this gene resulted in ovarian development in XY genotypes, qualifying it as the candidate MSD gene. Of note, in the X-linked *dmrt1*, a transposon inserted in intron 4 between two *cis*-regulatory elements. This structural change is connected to a shift of the epigenetic profile throughout the genomic region and down regulation of *X-dmrt1* before gonad differentiation. These results imply that transposon insertion in *X-dmrt1* is associated with the evolution of the SD system in the fighting fish. Our data provide novel insights into the origin of a MSD gene, as well as the evolution of the initial stage of differentiation of sex chromosomes in teleosts.

## Results

### A major locus determines sex while being affected by environmental factors

**Fig. 1:**
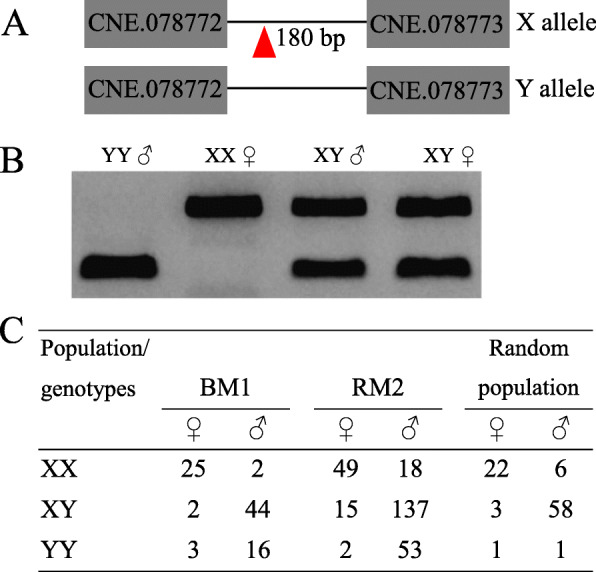
Association between genotypes and phenotypic sex at an InDel marker in sex determination locus of fighting fish. **A** Schematic drawing of the 180-bp insertion into the *X-dmrt1* locus between two predicted conserved noncoding elements. **B** PCR assays of males and females with different sex chromosome genotypes using the 180-bp InDel marker. **C** associations between genotypes and phenotypic sex at the InDel marker within F2 populations (BM1 and RM2) and a randomly collected population

First, we studied the mechanism of sex determination using selective crossings and genomic tools. After examining a series of test crosses for sex segregation, we obtained one family, P_xx×xy showing a female to male (M: F) ratio ≈1:1 (*n* = 25 M: 22F) in progenies. Genome scan with ~ 40 k SNPs from RAD sequencing for a SD locus in this cross identified only one major SD locus on LG2 (Additional file 1: Fig. S1A). We then examined associations between genotypes and sex phenotypes. All females were homozygous at one of the most differentiating SNPs (LG2: 2,081,899) while all males were heterozygous, indicating that the fighting fish has a XY SD system in the analysed population (Additional file 1: Fig. S1B, C and Table S1). To verify this hypothesis, we examined two additional F_2_ families: BM1 and RM2, which were independently set up by selecting a pair of F_1_ fish (Additional file 1: Fig. S2A). The whole pedigree of this population from parental (P) to F_2_ generations were raised under normal laboratory conditions and had been previously used for mapping fin and pigmentation related traits [23]. The F_1_ population showed a sex ratio of F: M ≈ 1:5 while the overall sex ratio across these two F_2_ families was F: M ≈ 1:2.8 (Additional file 1: Fig. S2A). We genotyped the parents of this pedigree using the above SNP marker. As expected, the P generation maternal and paternal fish were diagnosed with XX and YY genotype, respectively, while all F_1_ progenies were XY, regardless of sex. Genome scan for the SD locus across the whole F_2_ population with ~ 100 k SNPs consistently identified the same major locus at LG2 (Additional file 1: Fig. S2B). The overall genotypic segregation at the same peak SNP (LG2: 2,081,899) across F_2_ families was XX: XY: YY ≈ 1: 2.1: 0.8 (Additional file 1: Fig. S2C), which did not deviate significantly (Chi-squared test, *P* = 0.31) from the expected segregation for F_2_ populations. Surprisingly, ~ 11.5% of individuals in the F_2_ population were inconsistent between genotypes and phenotypic sex (Additional file 1: Fig. S2C), suggesting sex reversal. We then analysed the resequencing data of the putative XX, XY and YY genotypes and screened for InDels in a ~ 150-kb region that are tightly linked with the SD locus. A 180-bp insertion was detected in the intronic region of *dmrt1* in the X-linked locus between two predicted conserved noncoding elements (CNEs), CNE.078772 and CNE.078773. The inserted sequence was used to develop a diagnostic PCR assay (Fig. 1A, B). We further genotyped 91 fish randomly collected from Thailand, Singapore, Malaysia, China, and Indonesia and found that ~ 11.0% were likely sex reversed considering a genotypic XY SD system (Fig. 1C). These data are consistent with previous studies that suggested natural sex reversal could be common in fighting fish and that genetic sex determination (GSD) is frequently influenced by environmental factors [21, 22]. However, other explanations are also possible such as different sex-determining systems in different populations, including polygenic sex determination [22].

Interestingly, in another P_xx×yy test cross (the parents had XX and YY genotypes) the sex ratio of progenies were F:M ≈ 1:1 (Additional file 1: Fig. S3A and Table S1). The sex ratio observed in this test cross could be explained by substantial sex reversal, but other explanations are also possible. Thus, this population provided an opportunity to examine other SD loci in the fish. As expected, *F*_*ST*_ analysis between males and females with ~ 70 k SNPs showed that no SNP within the above major SD locus was associated with the phenotypic sex (Additional file 1: Fig. S3B). However, we detected some evidence of elevated *F*_*ST*_ peaks across several other chromosomes, e.g. LG1, LG10, LG12, and LG19 (Additional file 1: Fig. S3C). The *F*_*ST*_ values of these peaks were less than 0.2 and notably lower than that of the major sex locus of ~ 0.4 at LG2. In particular, these minor *F*_*ST*_ peaks varied across the three populations for test crosses (Additional file 1: Fig. S1-S3).

Sex is also likely affected by both abiotic and biotic environmental factors, e.g. temperature and social behaviours in some fish species [24, 25]. It is observed that temperature fluctuation could lead to sex ratio variation or sex reversal [26]. To examine whether sex was affected by temperature in fighting fish, three families were generated by crossing XX females and XY males and cultured at 23–25 °C for 1 month after fertilization, which is slightly lower than the common experimental temperature (~ 28 °C). At the time of sexual maturity, we observed that almost all fish were females (159 out of 160). In comparison, progenies generated by the same parents as the above showed a sex ratio of F: M ≈ 1: 1 (44 F: 39 M) at the time of sexual maturity when constantly cultured at ~ 28 °C. Admittedly, there might still be some other environmental factors that could likely influence the sex ratio.

In summary, our data provided genomic evidence that the fighting fish has a XY SD system in the analysed populations [21, 22]. At the same time, our data also explain sex ratio deviation beyond a stringent XY system and that both environmental factors, e.g. temperature, and minor-effect loci have the potential to modulate or even override the basal genetic sex determination of fighting fish.

### *Dmrt1* is the candidate MSD gene

**Fig. 2:**
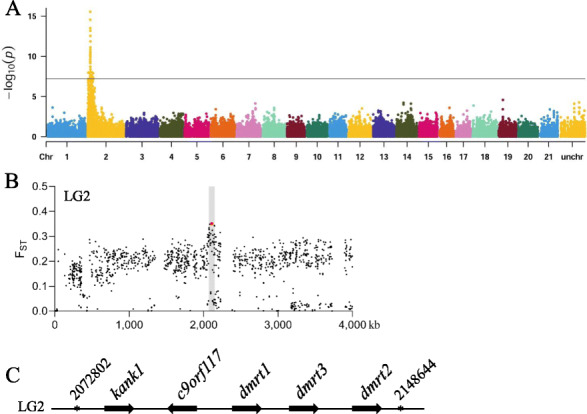
Mapping of the sex determining locus in fighting fish. **A** genome-wide association study for a sex determining locus based on all mapping populations, where the genome-wide significant cutoff value after Bonferroni corrections is shown (black horizontal line). **B** F_ST_ scan for sex determining locus on LG2 based on all mapping populations, where the 180-bp InDel marker is denoted with red. **C** annotation and genomic organization of protein coding genes in the peak genomic region of ~ 80 kb significantly associated with sex

**Fig. 3:**
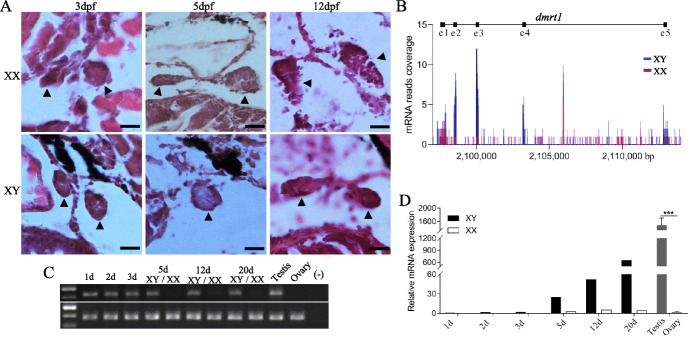
Differentiation of gonads of XX and XY genotypes and male-biased expression of *dmrt1* in fighting fish. **A** Gonad differentiation of XX and XY genotypes at 3, 5 and 12 days post fertilization (dpf), where scale bars indicate 10 μm. **B** Normalized coverage of mRNA reads mapped onto the *dmrt1* locus, obtained by sequencing transcriptomes of pooled XX and XY genotypes at 3 dpf, where coding regions are shown above the graph in the black horizontal line. **C** Expression of *dmrt1* in embryos and hatchlings of fighting fish at 1, 2, 3, 5, 12, 20 dph, and in gonads of adult male and female, analysed by reverse transcription PCR with gene-specific primers. House-keeping gene, β-actin, was used as positive control. Embryos with XY genotypes at 1, 2, and 3 dph were pooled for detection, while the other samples at 5, 12, and 20 dph were separately pooled for XX and XY genotypes. **D** Relative expression of *dmrt1* in the same samples as in **C**, examined using quantitative real-time PCR. Expressions in mature gonads were quantified in individual samples (*n* = 3). Difference between males and females was examined using Mann-Whitney test (***, *P* < 0.001)

To identify the MSD gene, a genome scan was done using 509 fish including 502 that were genotyped by RAD sequencing and seven additional fish that were re-sequenced from another study (Additional file 1: Table S1) [23]. Among these fish, 413 were from the above mapping families or shared the same genetic background with these families, while the remaining 96 were collected from brood populations in Asian countries, showing either fin or coloration related ornamental traits. Both the *F*_ST_ scan and GWAS, which takes genetic structure into consideration, consistently identified a peak genomic region of ~ 80 kb at LG2, showing the highest probability as MSD locus (Fig. 2A, B). Annotation of this region revealed five protein coding genes: *kank1*, *c9orf117*, *dmrt1*, *dmrt3a*, and *dmrt2* (Fig. 2C). Prior to examination of gene expression, we studied the gonad differentiation during development. The gonads were undifferentiated or not considerably differentiated at 3 and 5 days post fertilization (dpf), while differentiated at 12 dpf between XX and XY genotypes (Fig. 3A), suggesting that the developmental stage before 12 dpf is the critical stage for sex determination. Expression patterns of the five genes were first examined in embryos at early developmental stages using RT-PCR. Two genes, *kank1* and d*mrt1,* showed significant expression in pooled XY embryos at 24 hpf, but only *dmrt1* showed a clear male-biased differential expression between XX and XY embryos (Additional file 1: Fig. S4A, B). Transcriptome sequencing of pooled XX and XY embryos at 3 dpf revealed that *dmrt1* has the clearest male-biased expression among these genes (Fig. 3B and Additional file 1: Fig. S4C). In addition, *dmrt1* consistently showed male-biased differential expression from early developmental stages, before gonadal differentiation, to sexual maturity (Fig. 3C, D), and in adult fish. Its expression was detected only in the testis (Additional file 1: Fig. S4D). Taken together, the expression pattern of *dmrt1* in fighting fish is consistent with presence of the Y chromosome copy of *dmrt1Y*, but not with homozygosity of the X-copy of *dmrt1*, indicating that *dmrt1* is a most reasonable candidate for the MSD gene in fighting fish.

**Fig. 4:**
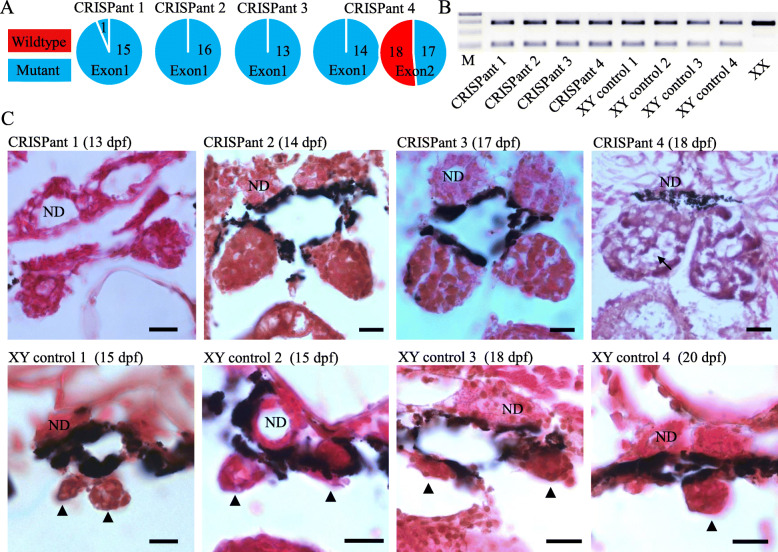
Knockout of *dmrt1* resulting in female development in fighting fish. **A** Proportion of wildtype and mutant alleles in the four screened CRISPants at both exon 1 and exon 2, where no mutant alleles were detected at exon2 for CRISPant 1, 2, and 3. **B** Genotypes of the four CRISPants, and four XY controls that showed no detectable mutant alleles at both exon 1 and exon 2, and XX control, determined by PCR assays using the 180-bp InDel in the SD locus. **C** Gonad differentiation of the four CRISPants at 13, 14, 17, and 18 dpf, respectively, in comparison to four randomly collected XY controls without detectable mutant alleles at 15, 15, 18, and 20 dpf, respectively, where arrow indicates the position of oocytes. Solid triangles indicate undifferentiated gonads. ND, nephric duct. Scale bars, 10 μm

To verify if *dmrt1* is the MSD gene in fighting fish, we knocked out this gene using the CRISPR/Cas9 system, with gRNAs targeting both exon1 and exon 2 (Additional file 1: Fig. S5). We screened four G_0_ XY fish with mutant alleles, which survived over 12 dpf when gonads have differentiated between sexes as revealed above. No wildtype allele was observed in three of the four G_0_ CRISPants at exon 1 and ~ 93.7 % (15 out of 16) of the alleles were mutants in the remaining CRISPant 1 (Fig. 4A and Additional file 1: Fig. S5). Histological examination showed that the gonad of CRISPant 1, at 13 dpf, had slightly differentiated from that of the XY control fish that were collected from 15 to 20 dpf and showed no evidence of mutant alleles (Fig. 4B, C). For the remaining CRISpants that were collected from 14 to 18 dpf, the gonads showed clear evidence of ovarian development (Fig. 4C). Particularly in CRISPant 4 at 18 dpf, the gonad was observed to have developed into an ovary with several oocytes (Fig. 4C). In summary, our data show that loss of function of *dmrt1* results in female development, suggesting *dmrt1* is necessary to drive male development in fighting fish.

We then examined the modes of gene duplication vs allelic diversification for the origin of the male determining *dmrt1* gene [3, 4, 7, 8]. We assembled the sequences of both the X and Y sex-linked region by sequencing the XX and YY P generation parents of the above F_2_ mapping population, and then separately mapped both XX and YY reads to the X and Y locus. If the MSD gene resulted from a recent gene duplication from an autosomal locus and translocation to the proto-Y creating the SD region, the sequence coverage for Y-specific reads to Y locus would be higher in the duplication region than the remaining genome. If the MSD gene resulted from recent local gene duplication in the current SD region, we would observe that the sequence coverage for Y-specific reads to the X locus is higher in the duplication region and also evidence of multi-mapped reads in the duplicated region of the Y locus. However, we found neither evidence of sequence coverage variation in the X or Y locus for both X-and Y-specific reads nor variation of distribution of multi-mapped reads along each locus (Additional file 1: Fig. S6A, B), indicating that the origin of the MSD gene *dmrt1* in fighting fish is not from gene duplication, but rather from allelic diversification.

### A transposon insertion in the X-linked *dmrt1* locus is associated with sex determination

**Fig. 5:**
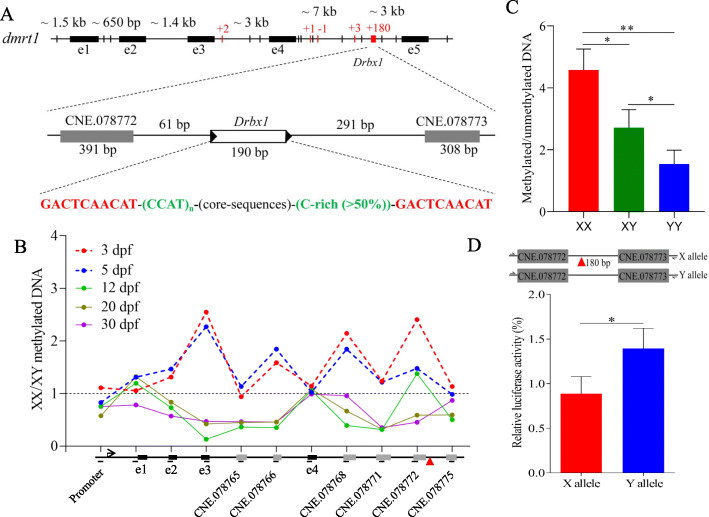
A transposon, *drbx1* inserted into the X linked *dmrt1* leads to epigenetic changes. **A** Genomic location of transposon *drbx1* in the X linked *dmrt1* locus and genomic feature of the transposon, where fixed variants, SNP and InDels, are shown with black and red vertical lines. For InDels, sequence variation in X allele relative to Y allele is denoted with positive and minus numbers for insertions and deletions, respectively. The transposon is characterized by 10-bp direct repeats at both ends. One copy of the 10-bp repeat element is also identified in the Y allele (representing the “target sequence duplication” of transposon insertion), leading to a 180-bp difference in length between X and Y alleles. The transposon is 61-bp and 291-bp, respectively, away from two predicted conserved non-coding elements (CNEs): CNE.078772 and CNE.078773. **B** Methylation levels of XX relative to XY pooled genotypes in predicted genomic elements including promoter, exon and conserved noncoding elements, throughout *dmrt1* genomic region, at 3, 5, 12, 20, and 30 dph, as examined using McrBC-based real-time PCR. Red arrow indicates the location of *drbx1*. **C** Relative methylation levels in the *dmrt1* promoter region of XX, XY, and YY genotypes at 3 dph, as examined using McrBC-based real-time PCR. Three independent pools for each genotype were analysed (Mann-Whitney test, *P < 0.05 and **P < 0.01). **D** The inverse relationship between methylation and expression for *dmrt1* at 5 dpf, examined based on pooled XX and XY genotypes. **E** Luciferase assay for transcriptional activation from the X and Y alleles. X allele containing both CNE.078772 and CNE.078773 and with the transposon *drbx1* insertion between the two CNEs was cloned into pGL3-Promoter vector and showed reduced luciferase activity in rainbow trout gonad cell line, in comparison to Y allele without the transposon insertion (Mann-Whitney test, *P < 0.05)

To understand how *dmrt1* determines sex in fighting fish, we analysed sequence variations between the X and Y SD loci, based on whole genome resequencing of XX, XY and YY genotypes. The variants identified within the genomic region of *dmrt1* were verified by Sanger sequencing. We found one missense SNP in the fifth exon of *dmrt1*, where three alleles were presented (922: G/A/T, Ala/Thr/Ser). However, sequencing of this position in the mapping populations showed that these alleles were neither fixed between sexes nor tightly linked with sex. In the non-coding sequences, the largest sequence difference was the 180-bp InDel that was used for genotyping above, while the remaining were randomly distributed SNPs and short InDels (Fig. 5A). Interestingly, this 180-bp InDel is an uncharacterized transposon, which we called *drbx1* (DNA repeat from *Betta splendens* on X chromosome 1), inserted in the X locus, located in the fourth intron of *dmrt1* (Fig. 5A). We genotyped this transposon in the mapping populations and found that this marker was completely linked with the peak SNPs in the genome scan (Fig. 2B). We searched the genome sequence of fighting fish (from a XX female with *drbx1* insertion in *dmrt1* [23]) using BLASTN (*E*-value < 1e−50 and coverage > 0.75) and found 553 copies of the core sequences of this transposon. Sequence structure and nucleotide composition analysis of these 553 copies belonging to the EnSpm-9_DR family in fighting fish showed that *drbx1* had a feature of 10-bp direct repeats at insertion sites and the core sequences flanked by both a simple repeat of (CCAT)n and a C-rich fragment with C content of > 50% (Fig. 5A).

Transposons can introduce epigenetic modifications to their flanking sequences [27]. We hypothesized that the insertion of the *drbx1* transposon has introduced epigenetic modifications to neighbouring *cis*-regulatory elements including but not limited to the *dmrt1* promoter. To verify this hypothesis, we examined the epigenetic variation throughout the genomic region that includes *dmrt1* using McrBC-based methylation assay. We observed that XX genotypes were higher methylated in comparison to XY genotypes in almost all examined coding elements and predicted CNEs throughout the whole genomic region that harbours *dmrt1* at 3 and 5 dpf, when the gonads are still not morphologically differentiated or only at the stage of initial differentiation, respectively. The methylation was found to spread beyond the insertion site of ~ 12 kb and reached the promoter region of *dmrt1* (Fig. 5B). We then examined the promoter region of different genotypes at 3 dpf, when the gonads are still undifferentiated, and found that the relative methylation level was significantly higher in XX than in XY. In YY fish, it was significantly lower than in the XY genotype (Fig. 5C). The overall methylation level throughout the genomic region presented an inverse relationship with expression for *dmrt1* at 5 dpf (Fig. 5D). Interestingly, the higher level of methylation in XX genotypes rapidly decreased, in comparison to the level of XY genotypes at 12, 20, and 30 dpf, when gonads are significantly differentiated between the two types of genotypes (Fig. 5B). These data indicate that the methylation status of the *dmrt1* genomic region, depending on the presence or absence of the transposon *drbx1* insertion, is correlated with genotypic sex at early developmental stages when gonads are still undifferentiated. It is lower for the Y-chromosomal allele of *dmrt1* and high for the X-chromosomal allele.

The *drbx1* transposon is located 61 bp and 291 bp away from two predicted conserved non-coding elements, CNE.078772 and CNE.078773 (Fig. 5A). We hypothesized that *drbx1* can affect the regulatory function of these two CNEs. We cloned the genomic element including both CNEs separately for X and Y allele (Fig. 5E) into a zebrafish enhancer detection (ZED) vector [28] and injected it into one-cell stage embryos of fighting fish. Both X and Y alleles showed enhancer activity detected as GFP fluorescence in the yolk at 48 hpf embryos when the gonads were undifferentiated (Additional file 1: Fig. S7). Bisulfite sequencing of the regions flanking transposon *drbx1,* using genomic DNA isolated separately from XX and YY genotypes at 3 dpf, when gonads are still undifferentiated, revealed that both CNE.078772 and CNE.078771 were higher methylated in the X allele than in the Y allele (Additional file 1: Fig. S8). We then examined if the *drbx1* insertion of the X locus affected the enhancer activity of the two neighbouring CNEs. The regulatory activities of both X and Y alleles were measured using Dual-Luciferase Reporter Assay. The Y allele showed significantly enhanced luciferase expression in rainbow trout gonad cell line compared to the X allele (Fig. 5E). Altogether, our data indicate that transposon insertion into the noncoding region of *dmrt1* introduces epigenetic and/or genetic changes that spatially affect the effects of both CNEs into the genomic region, leading to alteration of expression patterns of this gene by *cis*-downregulation of the X-allele during the critical stages of sex determination. Such a mechanism could explain the sex-biased expression of *dmrt1* during the sex determination period. However, there are other SNPs and short InDels in the genomic region of *dmrt1* and throughout the entire candidate SD locus of ~ 80 kb. Thus, we cannot exclude the possibility that there are further mutations in distant *cis*-regulatory elements that have additional roles in sex determination in fighting fish.

### X/Y divergence marks incipient sex chromosome evolution

**Fig. 6:**
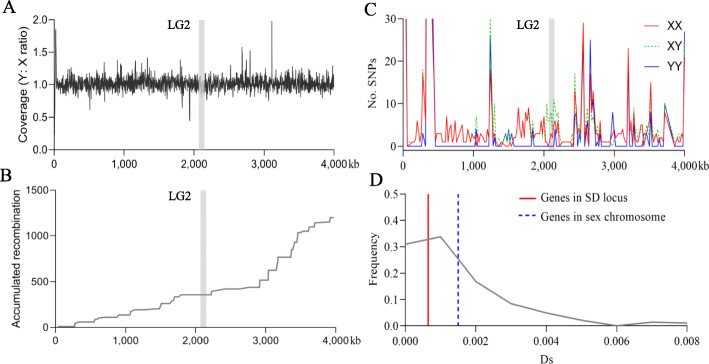
Restricted differentiation between X and Y chromosomes in fighting fish. **A** Ratio of sequencing reads coverage of Y-specific to X-specific reads mapped onto linkage group 2 (LG2) of the reference genome with XX genotype. The sex-specific region is highlighted with grey. **B** Accumulation of recombination rates along sex chromosomes, estimated based on high-density linkage map constructed using RM2 family. **C** Comparisons of the number of SNP within 5-kb non-overlapping windows, among XX, XY, and YY genotypes, based on whole genome resequencing data. **D** Distribution of Ds values of protein coding genes within the sex determining locus and sex chromosome, against the background (grey line)

To study sex chromosome differentiation, we assessed chromosome divergence by mapping X- and Y-specific reads to the XX female reference genome and investigated SNP density throughout chromosomes. If the Y has diverged from the X chromosome due to restricted recombination between X and Y chromosomes, we expected both the overall sequence coverage and SNP density for YY fish to be lower than those for XX fish. We found that the sex chromosomes were not differentiated both in terms of SNP density and sequence coverage (Additional file 1: Fig. S9A). In line with this result, we also did not find any difference in sequence coverage between X and Y chromosomes including the sex-specific region (Fig. 6A). For differentiated sex chromosomes, one significant signature is suppression of recombination [29–31]. We estimated recombination rates along individual chromosomes and found no evidence that recombination of sex chromosomes was lower in comparison to autosomes (Additional file 1: Fig. S9B). However, within the sex chromosomes, the sex specific region of ~ 80 kb was located in a small region with no recombination in the RM2 family that was generated by crossing XY × XY parents. Interestingly, this region spreads out to both sides and reaches up to ~ 370 kb (chr2: 1.83–2.20 Mb) (Fig. 6B). We further constructed sex-specific recombination maps and found no considerable difference between female and male-specific maps for the sex chromosome (57.55 vs 58.47 cM, respectively). Thus, our dataset does not inform on the presence or absence of heterochiasmy. Due to the lack of suitable samples, we were not able to directly compare the recombination rate between XX and XY parents. As recombination affects genetic diversity [32], we examined the genetic diversity in terms of the number of SNPs within 5-kb nonoverlapping windows for the pair of sex chromosomes based on whole genome sequencing data and found that XY genotypes showed higher SNP density than either XX and YY genotypes, but only in a limited region of ~ 200 kb surrounding the sex specific region (Fig. 6C). We verified this result in male and female populations using RAD sequencing data, and consistently observed higher nucleotide diversity in males compared to females in a short region of ~ 200 kb (chr2: 2.00–2.20 Mb) around the SD locus (Additional file 1: Fig. S9C). Finally, we found that Ds (synonymous substitution rate) values of protein coding genes either over the whole sex chromosome or in the sex-specific region did not obviously deviate from the background (Fig. 6D). Taken into further consideration that a heterogametic sex system is sustained by balancing selection that is highlighted by elevated Ds at the SD region compared to background [17, 33, 34], these results also support that the sex chromosomes of fighting fish have not diverged to a measurable level.

We separately genotyped the transposon *drbx1*, a candidate driver for the emergence of the SD system, in six wild *B. splendens* including two females and four males, and a pair of female and male *B. smaragdina* and *B. imbellis* that diverged from *B. splendens* within the last five million years (Additional file 1: Table S1) [35]. All these species belong to the *Betta splendens* complex [36, 37]. Interestingly, we found that *drbx1* is not present in the *dmrt1* locus of both *B. imbellis* and *B. smaragdina*. In the six wild *B. splendens,* the presence of *drbx1* noticeably deviated from the expectation of association with sex, in contrast to the situation in most domesticated fish (Additional file 1: Fig. S10) [35].

## Discussion

### The fighting fish MSD gene evolved by allelic diversification

Many studies have shown that genotypes are highly concordant with phenotypic sex in some fish species with a MSD gene [3, 4, 38], while in many others, sex reversal is very common [39–42]. In the present study, we found that the sex of most commercial stocks of fighting fish is determined by a major locus and could frequently be reversed. Minor additive effects from other loci influenced by environmental factors and discordance of phenotypic sex from the XY sex chromosome system were observed. In species with a polygenic SD system, sex ratio distortion varies across the given sex determining loci, due to independent segregation and a combination of additive and epistatic effects at these loci [25, 43]. However, whether these minor genetic loci in combination with environmental influences are responsible for the sex reversal in fighting fish is still not clear. It should be noted that we found one non-fixed SNP in the candidate MSD gene, *dmrt1* in the fighting fish. However, in fugu and *Seriola* fishes, a single amino acid substitution between the X and Y allele is sufficient to affect the signalling pathway of sexual development [4, 5]. Thus, it might be essential in the future to check whether the non-fixed missense SNP in the fifth exon of *dmrt1* affects sex ratio. In addition, we cannot exclude the possibility that there are different MSD genes in different populations of fighting fish. Artificial selective breeding between them might lead to one MSD gene dominating over the other when coexisting in a single individual, like in a XY/ZW hybridization system [43, 44]. Unfortunately, we were not able to determine the exact SD system of the test cross P_xx×yy. It is likely caused by the loss of Y chromosome in this specific ornamental lineage, reminiscent of the situation reported for the laboratory strains of zebrafish [45]. It is also possible that there are alternative sex determining mechanisms, including an ancestral state, which was devoid of Y, in this commercial stock. Previous studies have provided a plethora of evidence that *dmrt1* can act as the primary male sex determination gene in a number of vertebrates, including teleosts, but not mammals [8, 46–48]. In some species, *dmrt1* as the sex determiner is derived from gene duplication and neofunctionalization, e.g. medaka [8, 9], the African clawed frog [49], and spotted scat (*Scatophagus argus*) [50], while in other species, its sex determining function likely derives from alteration of expression patterns and efficiency of activation of downstream signalling pathways, due to allelic diversification, e.g. in frog (*Rana temporaria*) [51] and large yellow croaker (*Larimichthys crocea*) [52]. The sex-specific region of fighting fish and its immediate surrounding shows conserved synteny across vertebrates [9, 50, 52], where *dmrt1* is likely the ancestral copy of MSD gene in the above species. In medaka, *dmrt1Y,* a duplicated copy of *dmrt1* from this conserved locus, translocated and evolved independently as the SD gene [8, 9]. In spotted scat, a local duplication of *dmrt1* within the same locus is suggested to have generated the MSD gene [50]. The single copy of *dmrt1* in fighting fish is suggested to be the MSD gene. Together with *dmrt1* of the large yellow croaker [52] it provides a well-documented case of allelic diversification as origin of a SD gene in teleosts. Future investigations on sequence variations within and around *dmrt1* will uncover the mechanism of sex determination in fighting fish.

### A transposon inserted in *dmrt1* is associated with sex

It is hypothesized that transposable elements (TEs) are not only involved in the rewiring of regulatory networks to adapt to rapid turnover of SD systems as *cis*-regulatory elements [53–55] but are also able to directly regulate the expression patterns of key SD players and drive the turnover of the SD systems [24, 27]. It is reported that a transposon-induced methylation of the promoter of a transcription factor *CmWIP1* suppresses expression and thereby brings about sex determination in a plant species, the melon *(Cucumis melo L.)* [27]. However, this pattern of SD system has not been discovered in animals.

In this study, we have identified a transposon *drbx1*, inserted into the intronic region of the X-linked allele of *dmrt1*. Our data indicate that from there it suppresses the expression of *dmrt1* from the X chromosome by epigenetic silencing at the critical sex determination stage. In an allelic diversification event, alteration of the expression patterns is a common evolutionary path to MSD genes. In the Luzon ricefish, *O. luzonensis*, alteration of the expression pattern of the sex determining gene *gsdf* due to mutations in the promoter region is suggested to explain the function of this MSD gene [7]. Thus, it is hypothesized that mutations affecting gene expression pattern by *cis*-regulation are likely to bring about novel MSD genes. Transposons are able to reduce the expression of nearby genes by transposon-mediated enhancer-silencing [56, 57]. Besides this mechanism, transposons can also introduce novel transcription factor binding sites and mediate *cis*-regulation of gene expressions [53, 58, 59]. Previous studies in medaka and sablefish (*Anoplopoma fimbria*) have shown that transposons are involved in sex determination and act as regulatory elements by transcriptional rewiring of the SD regulatory network during the evolution of novel master SD genes [53–55]. Thus, transposons can play vital roles not only in driving differentiation between a pair of sex chromosomes [13, 60, 61] but also in the transitions of SD systems and turnover of sex chromosomes [27]. However, DNA methylation profile of key SD genes is vulnerable to environmental factors [24, 62, 63]. It is likely that sex determination caused by transposon silencing mechanism tends to be vulnerable, leading to variations of sex ratio. It is tempting to speculate that this explains the sex-reversals observed in fighting fish and on a broader level also in other species where environmental influence on a basic GSD mechanism is leading to discordance between genetic and phenotypic sex. It should mention that the YY females shown in Fig. 1C do not have the *drbx1* transposon inserted into *dmrt1*. The ratio of sex reversal for XX, XY and YY genotypes in the pedigree are 0.213, 0.086, and 0.068, respectively. Therefore, the YY genotypes without *drbx1* insertion have the smallest ratio of sex reversal, while XX genotypes with two copies of *drbx1* have the largest ratio of sex reversal. Further taken into consideration that the described sex determining mechanism is of recent origin and probably weak, superimposed to one or several ancestral sex determining mechanisms, and affected by environmental factors, the slight mismatch between YY genotypes and phenotypic sex is expected. It is possible that the *drbx1* insertion plays a major role in SD, while there are other variants that have minor effects on SD and lead to sex reversal in a very small proportion of fish. Knocking out *drbx1* and its flanking CNEs would provide more informative support to our hypothesis. Unfortunately, we did not manage to obtain CRISPants that carry deletions in these genomic elements, due to the complexity of microinjection manipulation in fighting fish [23] and also possibly that methylation around the transposon has reduced the efficiency of CRISPR/Cas9 system.

### Incipient differentiation between sex chromosomes

In the fighting fish, we showed that allelic diversification of *dmrt1* sparked the evolution of a MSD gene and promoted the formation of proto-sex chromosomes. We observed that the SD locus is limited to a very small region of ~ 80 kb, with signature of suppressed recombination and elevated sequence divergence. The sequence divergence in the coding regions of the SD locus on the X and Y chromosomes, however, is very low and only one nonfixed SNP was detected in the candidate MSD gene, *dmrt1*. There was no evidence of balancing selection in the genes within the SD locus. In particular, as revealed by the evolution of *drbx1*, this SD system is likely absent in the wild fighting fish and probably in other commercial stocks. These data suggest that the MSD gene in *B. splendens* has a recent origin and possibly arose during domestication within several hundred years [21], with gradual accumulation of sequence variation around *dmrt1* or the insertion site of *drbx1*. This situation is similar to the observations made in hybrid strains of swordtail fish (*Xiphophorus*) and papaya (*Carica papaya*), where the SD system turned over due to repeated hybridization and selection during domestication [64, 65]. Future comprehensive studies focussing on SD systems in large wild populations will provide valuable insights into the evolutionary history of the SD system of the studied species.

In the scenarios where sex chromosomes originate from duplication, translocation, and neofunctionalization of a MSD gene, recombination due to the ab initio lack of homology is suppressed and supposed to drive differentiation between sex chromosomes [31, 66]. In sharp contrast, as shown in fugu and *Seriola* fish species, MSD genes that emerge from allelic diversification of a single SNP do not necessarily undergo evident recombination suppression around the SD locus. Thus, those sex chromosomes are not progressively differentiated and remain conserved even over millions of years [4, 5]. We observed in the fighting fish that genetic changes leading to the emergence of the SD system are restricted to a small region, but can be of considerable size, clearly different from the above cases where MSD genes originated from a single SNP and showed no evidence of sequence divergence and recombination suppression [4, 5]. The MSD gene of fighting fish likely originated by an insertional mutation. Sequence divergence brought about by recombination suppression has not progressively spread, due to the young age of the MSD system in fighting fish. It should be noted that intrinsic genetic features of the genome sequences, e.g. GC content, selective sweeps, and rapid lineage sorting each can independently lead to divergence of chromosomes [67, 68]. In particular, DNA methylation is also suggested to epigenetically silence recombination [69, 70].

We verified that some commercial stocks of the fighting fish have a X/Y SD system, with frequent sex reversal. The MSD locus is located in a small region at LG2, where it shows limited differentiation between the pair of sex chromosomes. Within the SD region, *dmrt1* showing a male-biased expression pattern and being necessary for male development is the candidate MSD gene, originating from allelic diversification. We showed that the transposon *drbx1* is located into the X-linked *dmrt1* locus. It most likely reduces the expression of *dmrt1* in females, explaining a male-specific function. *dmrt1* is known to be a dosage sensitive male determining transcription factor. Haploinsufficiency of *dmrt1* in humans results in male to female sex reversal [71]. In birds and the Chinese tongue sole, compromised expression of one copy of *dmrt1* leads to female development [46, 48]. In these organisms, the chromosome with the inactive *dmrt1* evolved to a W chromosome thereby ensuring higher levels of *dmrt1* expression in the homogametic ZZ genotypes and consequently male development. Also, in the frog *Xenopus laevis*, the chromosome, which harbours a mutant version of *dmrt1* (DM-W) that is responsible for lowering *dmrt1* functional availability for female development [49], is a W chromosome. In this respect, the here proposed mechanism for fighting fish provides a so far undescribed solution to the same objective of lowering *dmrt1* expression: the proto-sex chromosome that might have arisen by a single genomic event, namely insertion of a transposon, became an X chromosome.

## Conclusions

In summary, we identified a transposon, *drbx1*, inserted into the fourth intron of the X-linked, but not Y-linked, *dmrt1* allele in domesticated fighting fish. *drbx1* reduces the enhancer activity of two nearby, closely linked *cis*-regulatory elements. Furthermore, *drbx1* is associated with hypermethylation around the insertion site that spreads to the *dmrt1* promoter. Together, this leads to downregulation of *dmrt1* expression by the time of gonad differentiation in XX fish and allows female sexual development. This mechanism represents a previously undescribed solution in animals relying on *dmrt1* function, which is the transposon-induced epigenetic regulation for sex determination ultimately resulting in a dosage effect of *dmrt1* activity between sexes. Our data provide evidence that the described sex chromosomes of fighting fish originated from a recent allelic diversification process but are still in the initial stage of differentiation, providing novel insights into the evolution of sex determination genes and sex chromosomes in vertebrates.

## Methods

### Populations for mapping the sex determination locus

Four test crosses of *B. splendens* were generated to examine phenotypic segregation of sex. For detailed information on the animals used in this study see Additional file 1: Table S1. The parental fish were randomly collected from commercial brood stocks and raised under normal laboratory conditions. A family, P_xx×xy, showing sex ratio of ~ 1:1 in progenies was first selected for mapping sex and development of sex-specific markers. The second pedigree showing XX and YY genotypes for P generation maternal and parental fish, respectively, and a ratio of females to males of ~ 1:3 for the F_2_ generation was selected for studying sex determination. This pedigree also showed F_2_-type segregation of both double-tail and melanic phenotypes and were used for mapping the two traits in another study [23]. Two F_2_ families, BM1 and RM2, were selected for examination of sex segregation. In addition, a family, P_xx×yy that was generated by crossing a XX-female and a YY-male parents based on sex-linked markers, was produced to examine naturally occurring sex reversal. Finally, 91 domesticated fish that were collected from ornamental brood stocks across Asian countries including Singapore, Malaysia, Thailand, Indonesia, and China were also used for genetic mapping of sex. Sex was determined by dissection of the gonads and macroscopic inspection of ovaries or testis of each fish.

### RADseq and whole genome sequencing

Fish from BM1 and RM2, consisting of 366 individuals, were genotyped using RADseq [72] in another project to study the genetic basis of fin shape and coloration phenotypes [23]. The remaining 136 fish including 47 from P_xx×xy and 89 randomly collected from Asian countries were sequenced using both the same RADseq protocol and with similar sequencing depth as in our previous study [23]. We further sequenced separately one putative XX female, XY male, and YY male each collected from Singapore to identify sex-linked markers and study sex chromosome evolution. Libraries of ~ 500 bp inserts were constructed using Illumina Truseq DNA PCR free Library Preparation Kit (Illumina). Libraries were sequenced as 150 bp paired-end reads on NextSeq500 (Illumina), and ~ 50× coverage of reads for each individual was obtained. Raw sequencing reads were cleaned with process_radtags in Stacks package [73] and then were aligned to the reference genome that we have constructed recently based on a female fish with XX genotypes at *drbx1* [23], using BWA-mem [74] with default parameters. SNP calling was carried out using the best practices workflows of Picard/GATK v4.0 [75]. Raw variants were filtered with the following parameters: ‘QD < 2.0 || FS > 60.0 || MQ < 40.0 || MQRankSum < -12.5 || ReadPosRankSum < -8.0 || SOR > 4.0’ and ‘QD < 2.0 || FS > 200.0 || ReadPosRankSum < -20.0 || SOR > 10.0’, for SNPs and indels, respectively. The obtained variants were further filtered with ‘--minDP 7, --max-missing 0.8, --maf 0.1’ using VCFtools [76], after which ~ 110 k variants were kept.

### Genome scan for sex-linked markers

We first carried out a genome-wide association study (GWAS) to map the sex locus, which considered the genetic background in the mapping populations. GWAS was performed with compressed mixed linear model implemented in the R package GAPIT [77], with population structure as a covariate. We also conducted *F*_ST_ based genome scans for sex locus with VCFtools [76]. We further developed InDel markers for fast PCR assays for sex by analysing whole genome sequencing data sets. In detail, putative XX and YY genotypes were de novo assembled using ABySS 2.0 [78] with default parameters. The shortest genomic region resulting from association mapping was used for marker screening. InDels were screened by both GATK pipeline [75] and manual alignment for long InDels between the two genome assemblies. Primers of these InDels of suitable length were then designed for validation using PCR assays.

### Gene expression analysis

Gonadal sex differentiation for samples from the P_xx×xy pedigree in early developmental stages was determined by histological examination (*n* > 4 for each genotype). Fish heads were dissected for DNA isolation and genotyping, while the trunks were fixed in 4% paraformaldehyde (Sigma) and embedded into paraffin (Roche). Serially cross-sectioned trunks (7 μm thickness) were stained with haematoxylin and eosin (HE) as described [79] to examine gonadal differentiation. Gene expression was examined in pooled genotypes by both reverse transcription PCR (RT-PCR) and mRNA sequencing. For RT-PCR and mRNA sequencing, total RNA was isolated using TRIzol reagent (Invitrogen). Two micrograms of total RNA were treated with DNase I (Roche) and then used for cDNA synthesis using Reverse Transcriptase M-MLV (Promega). RT-PCR followed a previously described protocol [9]. In brief, cDNA from 500 ng of total RNA extracted from 40 pooled genotyped embryos/trucks were used for reactions with gene-specific primers and housekeeping gene (Additional file 1: Table S2). For adult tissue samples, cDNA from 20 ng of total RNA was used. Standard PCR was carried out with 35 cycles at annealing temperature of 60 °C. Expression was also examined using real-time RT-PCR (qRT-PCR), using KAPA™ SYBR® FAST qPCR Kits (Kapa Biosystems) with CFX96 Touch™ Real-Time PCR Detection System (Bio-Rad). Beta actin was used as reference. Three replicates were performed for each sample and the 2^−ΔΔCT^ method was used to quantify the relative gene expression. Libraries for mRNA sequencing were constructed using TruSeq RNA Library Prep Kit v2 (Illumina) and sequenced on NextSeq500 (Illumina) for 2 × 150 bp paired-end reads (~ 48 M reads for each genotype). Raw reads were cleaned with process_shortreads in Stacks package [73] and mapped to reference genome with STAR [80], with default parameters. HTSeq-count [81] was employed to quantify transcripts for each sample and transcripts were normalized by Transcripts Per Kilobase Million (TPM) for comparison between samples.

### Methylation assay and bisulfite sequencing

Methylation profiles of the genomic fragment of interest were analysed using a protocol based on methylation-sensitive restriction enzymes [27]. Total genomic DNA from the trunks of embryos was digested overnight using McrBC (NEB) with a parallel control without digestion. DNA of 15 ng from both digested and undigested was used for quantification of the relative methylation level using DNA element-specific primers (Additional file 1: Table S2). A single-copy genomic region (chr1:14737421-14737671) without CG site and showing no difference between digested and undigested treatments when using equal total DNA for quantification using qPCR, was used to normalize all samples. Estimation of methylation levels was calculated as the ratio of methylated to non-methylated DNA with the formula 2^ΔCT^ according to a previous study [27]. For bisulfite sequencing of DNA elements of interest, 500 ng genomic DNA isolated from the trunks of embryos of different genotypes were treated with EZ DNA Methylation Kit (Zymo Research) for bisulfite conversion. Primers were designed using MethPrimer [82] and PCR was carried out using EpiMark Hot Start Taq DNA Polymerase (NEB, USA). Methylation frequency of cytosine in the target DNA fragment was estimated by sequencing 96 clones.

### Enhancer reporter assays

To verify enhancer activity of *cis*-regulatory element, the X and Y alleles, including the flanking predicted conserved noncoding elements, CNE.078772 and CNE.078773 (Fig. 5D), were cloned into pGL3-Promoter vector (Promega). The constructs were then separately transfected into RTG-2 rainbow trout gonad cell line (https://www.atcc.org), together with the Renilla Luciferase Control Reporter Vector (Promega), using Lipofectamine 3000 (Thermo Fisher), with three replications. After 48 h post transfection, luciferase activity was measured using Dual-Luciferase Reporter Assay System (Promega). Putative enhancer sequences were also constructed into the Zebrafish Enhancer Detection (ZED) Vector [28]. ZED constructs of 40 ng/μl and T7-Transposase (Addgene no. 51818) mRNA of 50 ng/μl that was transcribed using mMESSAGE mMACHINE T7 kit (Life Technologies) were co-injected into one-cell stage embryos. Expressions of reporter GFP and internal control RFP were imaged using a Leica MZFLIII microscope.

### Knockout *dmrt1* using CRISPR/Cas9

We introduced mutations in the coding sequences of *dmrt1* with CRISPR/Cas9 system according to our previous study [23]. In brief, three guide RNAs (gRNAs), two with targets on exon 1 and one on exon 2, were designed using E-CRISP [83]. No off-targets were detected in the reference genome for the three gRNAs. Templates of gRNAs were synthesized with flanking common adapters: 5′-TAATACGACTCACTATA[GGN(20)]GTTTTAGAGCTAGAA-3′. A universal primer (5′-AAAAGCACCGACTCGGTGCCACTTTTTCAAGTTGATAACGGACTAGCCTTATTTTAACTTGCTATTTCTAGCTCTAAAAC-3′) was then used to assemble gRNA templates with PCR using Q5® High-Fidelity DNA Polymerase (NEB). PCR products were cleaned with QIAquick PCR Purification Kit (Germany) and 150 ng purified DNA were transcribed using HiScribe T7 High Yield RNA Synthesis Kit (NEB). gRNA products were then purified using miRNeasy Mini Kit (Qiagen). One nanoliter of Cas9 Nuclease NLS proteins (200 ng/μL) (NEB) and gRNAs (total, 200 ng/μL) was co-injected into each of the one-cell stage embryos that were generated by crossing XX female and XY male parents, and over 2000 embryos were injected. All injected embryos were cultured under normal laboratory conditions. Mutations in the injected embryos were screened by T7 endonuclease-based detection with EnGen® Mutation Detection Kit (NEB). Genetic modifications were further confirmed by TA cloning and Sanger sequencing of the DNA fragments spanning the targets of gRNAs with locus-specific primers. Gonads of both XY mutants and controls that showed no detectable modifications in the coding sequence of *dmrt1* with targets of gRNAs were examined by histological cross-sections as described above.

### Evolutionary analyses of sex chromosome divergence

As the families used for RADseq were either generated by crossing putative XY male and XY female or sequenced without parental samples, we could not directly compare recombination rates between a pair of XX and XY chromosomes. We estimated the recombination rates along sex chromosomes based on the sex averaged linkage map constructed by using the RM2 family data from our previous study [23]. Recombination fractions between adjacent marker pairs were estimated and mapped along the physical map in cM/Mb, according to a previous study [84]. Accumulated recombination rates along chromosomes were then used to compare the relative recombination rates between adjacent genomic regions. For comparison of recombination rates among chromosomes, we estimated variation of recombination rates with the program LDhat [85]. Using whole-genome sequencing data sets of different genotypes, we estimated sequencing coverage along individual chromosomes to study sequence divergence between putative X and Y chromosomes. Cleaned reads were mapped to the reference genome with BWA-MEM [74] with default parameters and sequence coverage was estimated by dividing the chromosome into windows of same size using BBTools (https://jgi.doe.gov/data-and-tools/bbtools/). Different window sizes were tested to examine the consistency among results. Finally, we annotated the genome of XX and YY genotypes and extracted CDS sequences. Pairwise alignments between CDS sequences were performed using the program MUSCLE [86], and Ds was calculated using the program KaKs_calculator [87]. Only CDS with length of no more than 3000 bp and no less than 300 bp, were retained for this analysis.

## Supplementary Information

**Additional file 1: Figures S1-S11** and **Tables S1 & S2**. **Figure S1.** Mapping of the sex determining locus in family P_xx×xy of fighting fish. **Figure S2.** Mapping of the sex determining locus in F2 populations including BM1 and RM2 families. **Figure S3.** Mapping of sex determining locus in a family, P_xx×yy, generated by crossing a putative XX female and a YY male. **Figure S4.** Male-biased expression of *dmrt1* in fighting fish. **Figure S5.** Knockout of *dmrt1* using the CRISPR/Cas9 system in fighting fish. **Figure S6.** Sequence coverage for X- and Y-specific reads on either the X or Y putative *dmrt1* containing the SD locus. **Figure S7.** Predicted conserved noncoding elements flanking transposon *drbx1* enhance reporter GFP expression. **Figure S8.** Difference in methylation profile in the genomic regions flanking the transposon *drbx1* insertion site between X and Y alleles. **Figure S9.** Restricted differentiation between X and Y chromosomes in fighting fish. **Figure S10.** Transposon *drbx1* is not associated with phenotypic sex in wild species of *B. splendens* complex. **Figure S11.** Original uncropped gels for figures and supplementary figures used in this study, where how the gels were cropped is indicated with red box. **Table S1.** Samples including commercial stocks and wild *Betta* fish used in this study. **Table S2.** Sequences of primers used in analyzing sex determination in the fighting fish.

## Data Availability

Sequences used for RAD, RNA, and genome sequencing are achieved in the DDBJ Sequencing Read Archive (SRA) database under BioProject ID: PRJDB7253- PRJDB7255 [23, 88].

## Abbreviations

CNEs: Conserved noncoding elements
dpf: Days post fertilization
*drbx1*: DNA repeat from *Betta splendens* on X chromosome 1
GSD: Genetic sex determination
MSD: Master sex determining
P: Parental
SD: Sex determination
TEs: Transposable elements
TGF-β: Transforming growth factor-β
