# Supplementary material for: Transposon-induced epigenetic silencing in the X chromosome as a novel form of dmrt1 expression regulation during sex determination in the fighting fish
Source: BMC Biol. 2022 Jan 7;20:5. doi: 10.1186/s12915-021-01205-y (PMC8742447; doi:10.1186/s12915-021-01205-y)
Supplement: Supplementary file 1 — Additional file 1: Figures S1-S11 and Tables S1 & S2. Figure S1. Mapping of the sex determining locus in family P_xx×xy of fighting fish. Figure S2. Mapping of the sex determining locus in F2 populations including BM1 and RM2 families. Figure S3. Mapping of sex determining locus in a family, P_xx×yy, generated by crossing a putative XX female and a YY male. Figure S4. Male-biased expression of dmrt1 in fighting fish. Figure S5. Knockout of dmrt1 using the CRISPR/Cas9 system in fighting fish. Figure S6. Sequence coverage for X- and Y-specific reads on either the X or Y putative dmrt1 containing the SD locus. Figure S7. Predicted conserved noncoding elements flanking transposon drbx1 enhance reporter GFP expression. Figure S8. Difference in methylation profile in the genomic regions flanking the transposon drbx1 insertion site between X and Y alleles. Figure S9. Restricted differentiation between X and Y chromosomes in fighting fish. Figure S10. Transposon drbx1 is not associated with phenotypic sex in wild species of B. splendens complex. Figure S11. Original uncropped gels for figures and supplementary figures used in this study, where how the gels were cropped is indicated with red box. Table S1. Samples including commercial stocks and wild Betta fish used in this study. Table S2. Sequences of primers used in analyzing sex determination in the fighting fish. [file 12915_2021_1205_MOESM1_ESM.docx]

**Supplementary information for the paper entitled**

**“Transposon-induced epigenetic silencing in the X chromosome as a novel form of *dmrt1* expression regulation during sex determination in the fighting fish”**

Le Wang^1#^, Fei Sun^1#^, Zi Yi Wan^1^, Zituo Yang^1^, Yi Xuan Tay^1^, May Lee^1^, Baoqing Ye^1^, Yanfei Wen^1^, Zining Meng^2^, Bin Fan^3^, Yuzer Alfiko^4^, Yubang Shen^5^, Francesc Piferrer^6,^ *, Axel Meyer^7,^ * Manfred Schartl^8,9^ * & Gen Hua Yue^1,10,11^ *

^1^ Molecular Population Genetics & Breeding Group, Temasek Life Sciences Laboratory, Singapore 117604, Singapore

^2^ School of Life Sciences, Sun Yat-sen University, Guangzhou 510275, China

^3^ Department of Food and Environmental Engineering, Yangjiang Polytechnic, Yangjiang 529500, China

^4^ Biotech Lab, Wilmar International, Jakarta, Indonesia

^5^ Key Laboratory of Exploration and Utilization of Aquatic Genetic Resources, Shanghai Ocean University, Shanghai 201306, China

^6^ Institute of Marine Sciences (ICM), Spanish National Research Council (CSIC), 08003 Barcelona, Spain

^7^ Department of Biology, University of Konstanz, 78457 Konstanz, Germany

^8^ Developmental Biochemistry, Biocenter, University of Wuerzburg, 97074 Wuerzburg, Germany

^9^ The Xiphophorus Genetic Stock Center, Department of Chemistry and Biochemistry, Texas State University, San Marcos, Texas 78666, USA

^10^ Department of Biological Sciences, National University of Singapore, Singapore 117543, Singapore

^11^ School of Biological Sciences, Nanyang Technological University, Singapore 637551, Singapore

^#^ Contributed equally to this study

^*^ Corresponding authors

FP: piferrer@icm.csic.es

AM: axel.meyer@uni-konstanz.de

MS: phch1@biozentrum.uni-wuerzburg.de

GHY: genhua@tll.org.sg


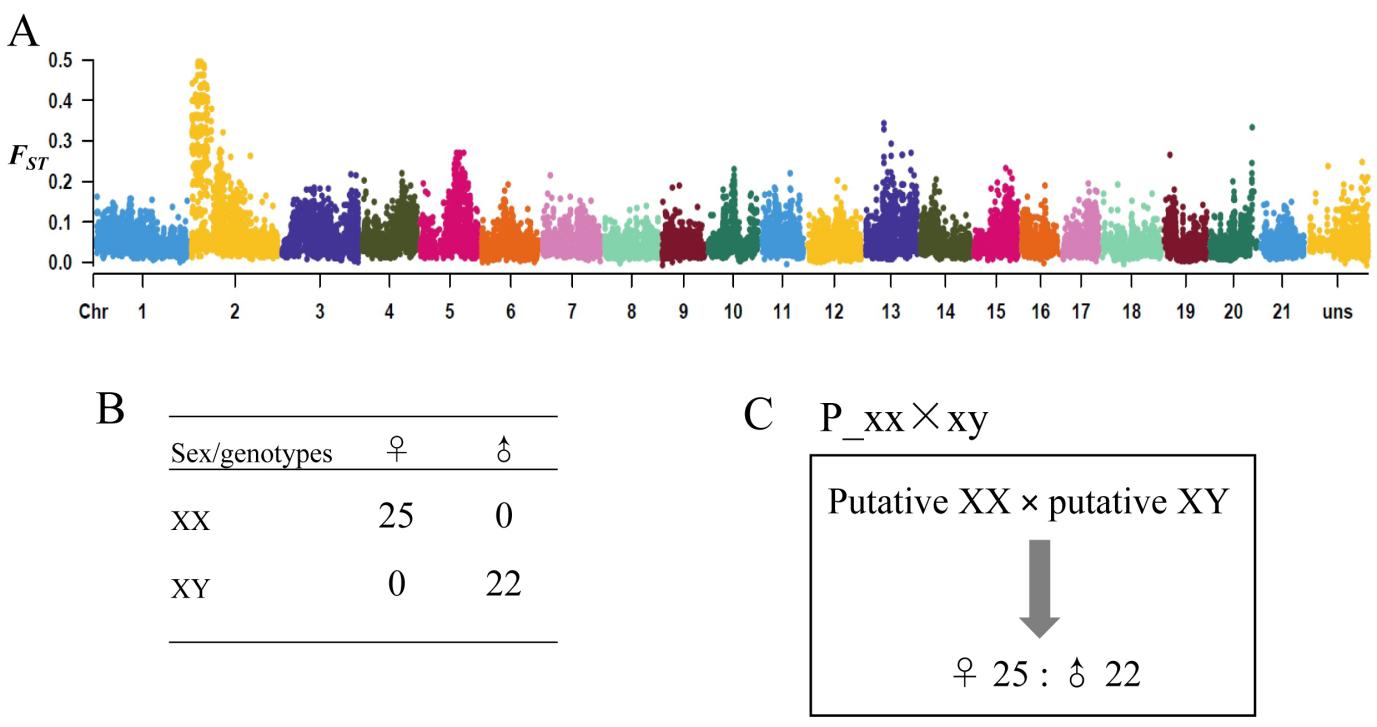


**Fig. S1** Mapping of the sex determining locus in family P_xx×xy of fighting fish. A, genome-wide *F_ST_* scan for the SD locus in this family, where a major peak was identified at LG2. B, the number of phenotypic female and male fish showing to be homozygous (XX) and heterozygous (XY), at the most differentiating SNP, LG2: 2,081,899. C, sex ratio of male to female in family P_xx×xy, inferring putative XX and XY genotypes for paternal and maternal fish.


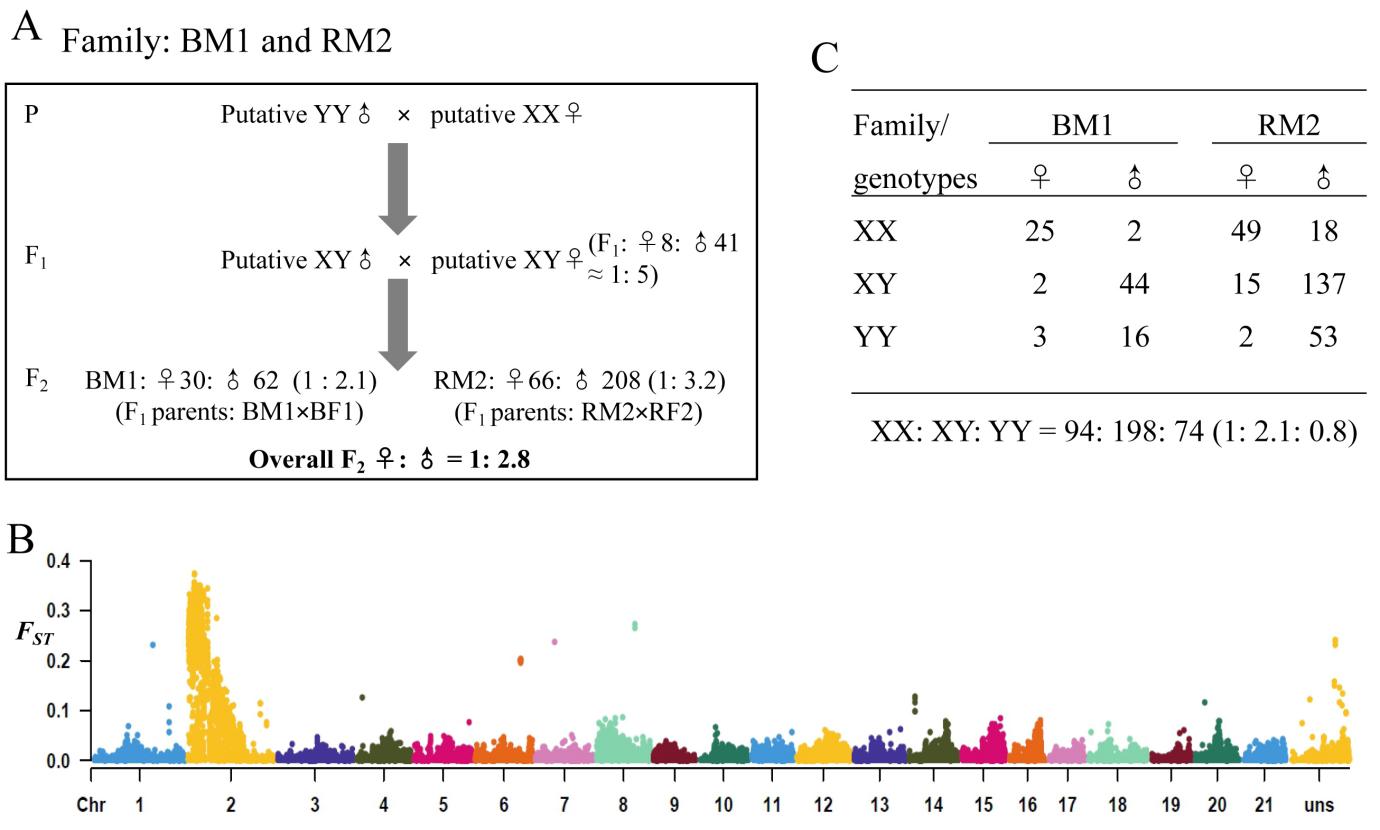


**Fig. S2** Mapping of the sex determining locus in F_2_ populations including BM1 and RM2 families. A, sex ratio of male to female in one F_1_ (8 females and 41 males) and two F_2_ families: BM1 and RM2, where inferred genotypes for parental fish are also indicated. B, genome-wide F_ST_ scan for SD locus across F_2_ families, where a major peak was identified at LG2. C, association between phenotypic sex and genotypes at the most differentiating SNP, LG2: 2,081,899, across the two F_2_ families.


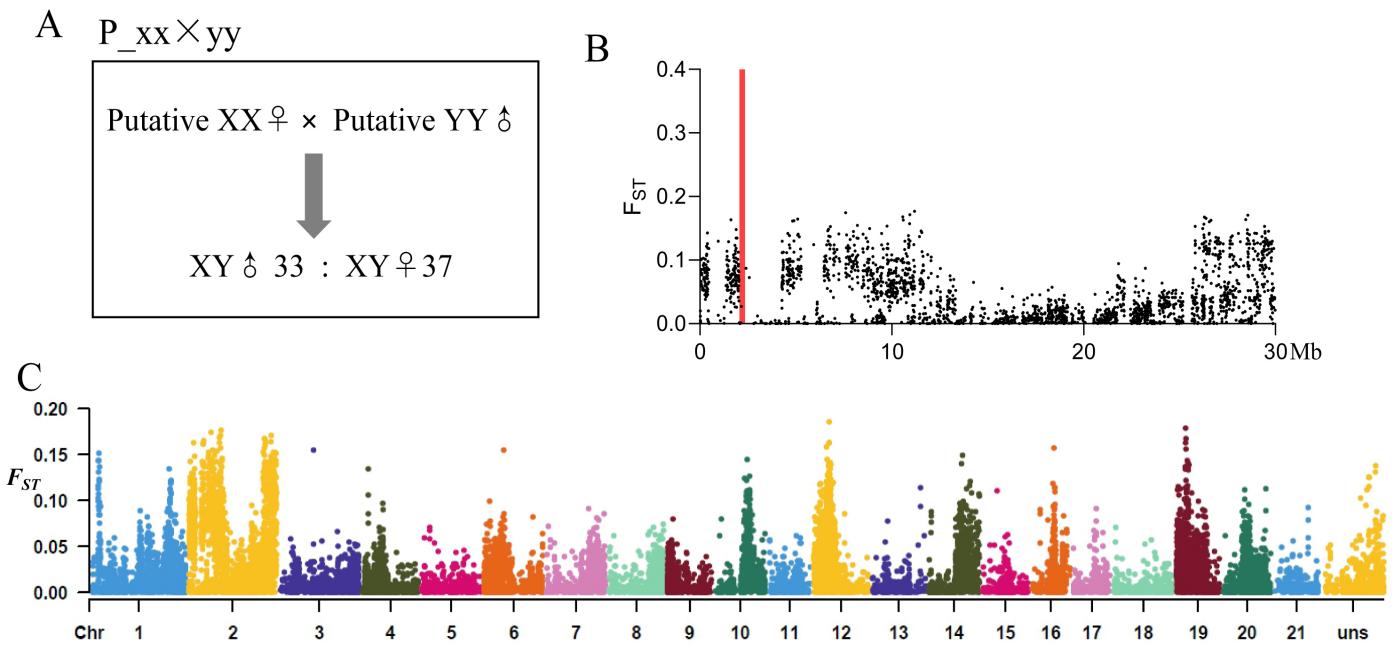


**Fig. S3** Mapping of sex determining locus in a family, P_xx×yy, generated by crossing a putative XX female and a YY male. A, sex ratio of male to female in the offspring of family P_xx×yy, all of which show putative XY genotypes. B, *F_ST_* scan throughout the sex chromosome shows that the master SD locus identified at LG2 in the other mapping populations, highlighted with red vertical bar, has no differentiation. C, genome-wide *F_ST_* scan for SD locus across family P_xx×yy, where the master SD locus identified at LG2 in the other mapping populations disappears while several minor-effect loci appear.


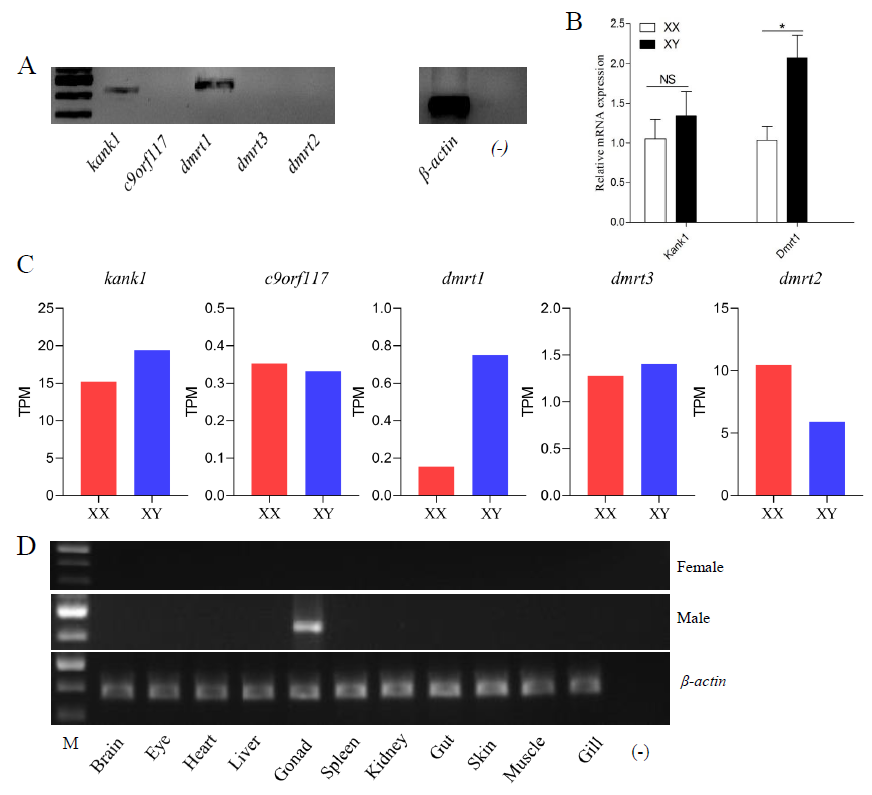


**Fig. S4** Male-biased expression of *dmrt1* in fighting fish. A, expression of predicted protein coding genes in the master SD locus, examined using RT-PCR based on RNA isolated from pooled XY embryos at 24 hpf. Expression of *β-actin* was used as positive control. B, Relative expression of candidate genes, *kank1* and *dmrt1*, in pooled embryos of XX and XY genotypes using reverse transcription real-time PCR (* denotes *P* < 0.05 with Mann-Whitney test; n = 3). Heads of embryos were used for DNA extraction, PCR and genotyping. Genotypes were determined using the 180-bp InDel marker. C, relative expression of protein coding genes in the candidate SD locus, examined by transcriptome sequencing of pooled XX and XY individuals, at 3 dpf, where *dmrt1* shows the most prominent male-biased expression. D, expression patterns of *dmrt1* in 11 tissues of adult male and female fish. House-keeping gene, *β-actin*, was used as positive control. Expression of *dmrt1* was only observed in the testis.


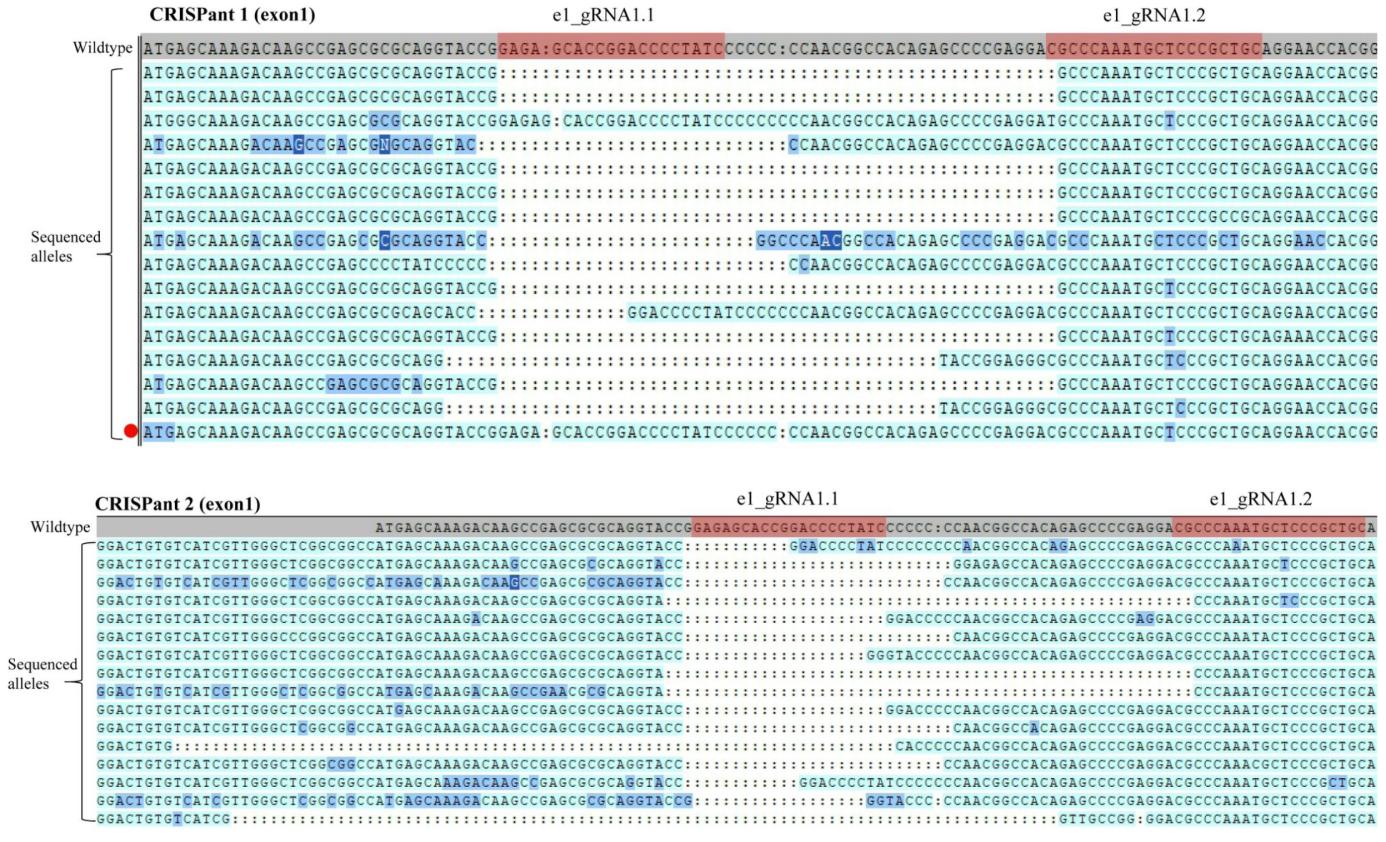


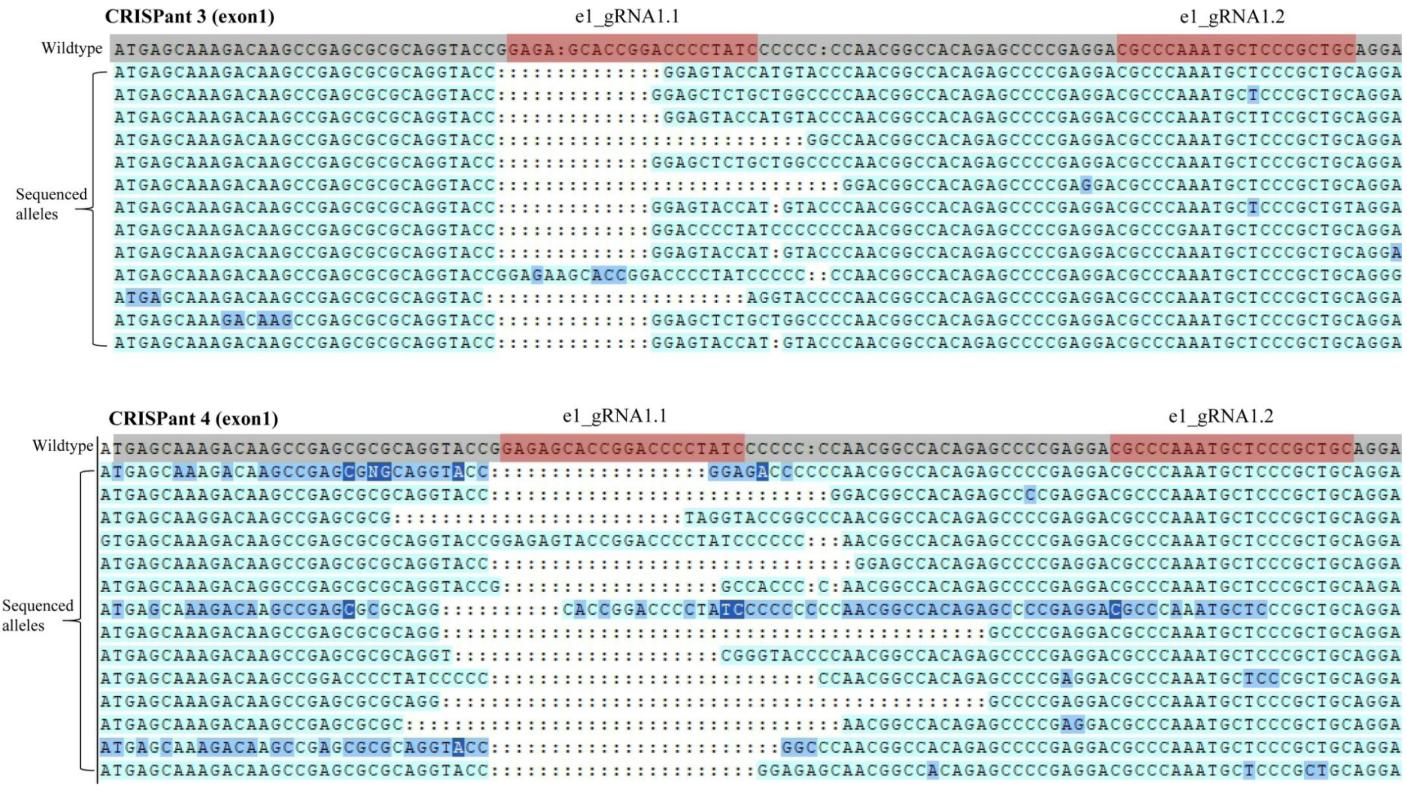


**Fig. S5** continued


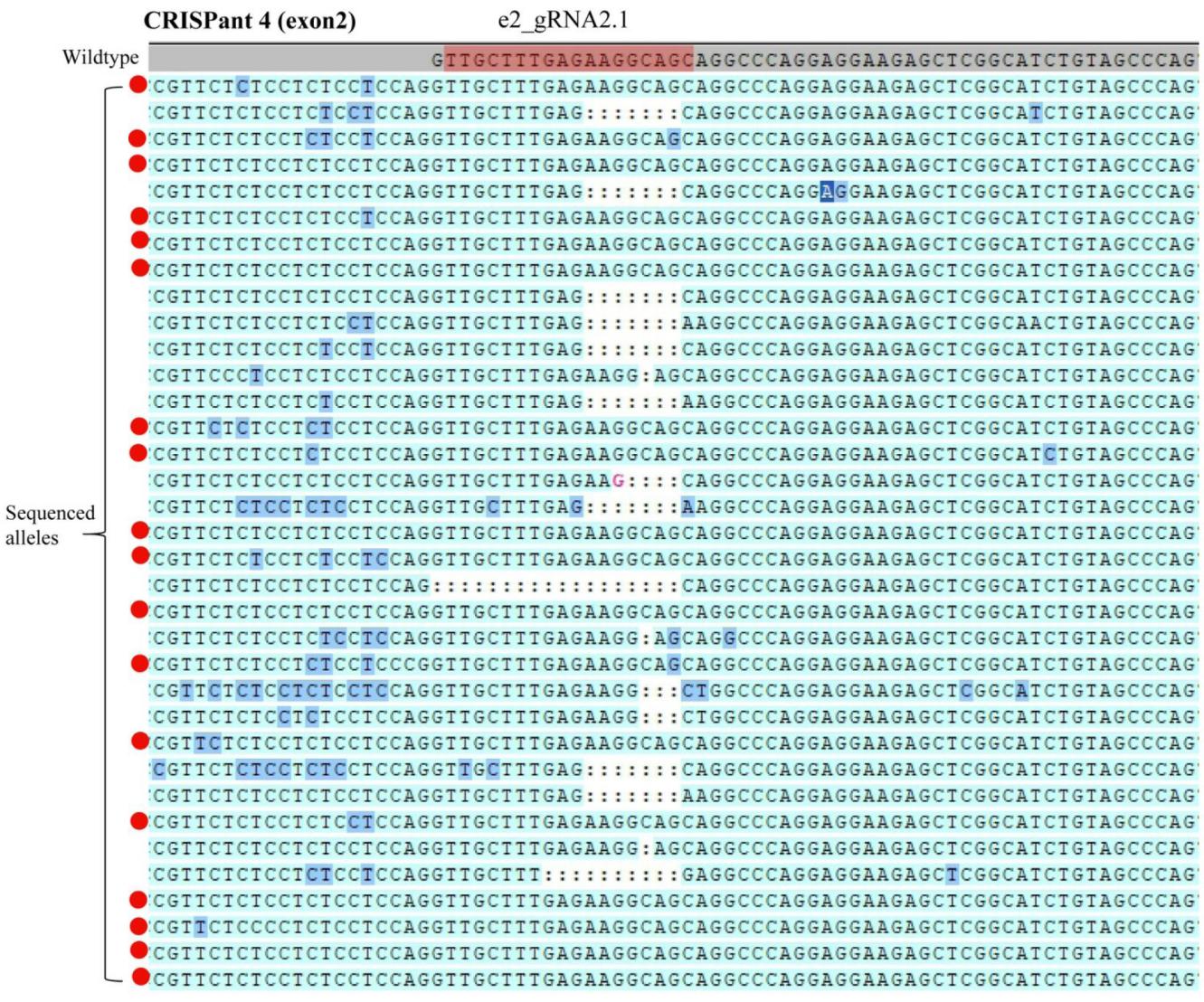


**Fig. S5** Knockout of *dmrt1* using the CRISPR/Cas9 system in fighting fish. Two guide RNAs (gRNAs): e1_gRNA1.1 and e1_gRNA1.2 were designed to modify exon 1 and one gRNA: e2_gRNA2.1 was used to modify exon 2 of *dmrt1*. The three gRNAs were co-injected together with Cas9 protein into one cell stage embryos. Four fish were identified to carry mutant alleles. DNA fragments spanning these target sites were amplified and sequenced following TA cloning. Sequence alignments show all four CRISPants that were modified at exon 1 and only CRISPant 4 was modified at exon 2. In the alignment, wildtype allele sequences were used as reference and unmodified allele sequences that were identified by sequencing are highlighted with solid red dot shown in the left. Positions of gRNAs are indicated in red shade on top of each alignment. The blue letter indicates the position has sequencing noise. This sequencing noise doesn’t affect the determination of the sequenced nucleotide acid.


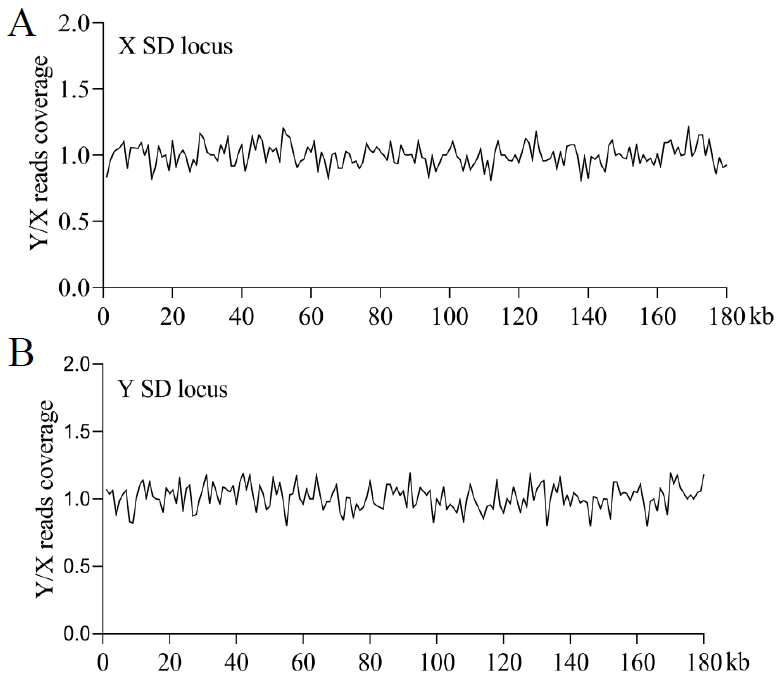


**Fig. S6** Sequence coverage for X- and Y-specific reads on either the X or Y putative *dmrt1* containing the SD locus. A, relative sequence coverage of Y-specific reads to X-specific reads in 1-kb sliding window, mapped to the X locus and its 50-kb flanking regions. B, relative sequence coverage of Y-specific reads to X-specific reads in 1-kb sliding window, mapped to the Y locus and its 50-kb flanking regions.


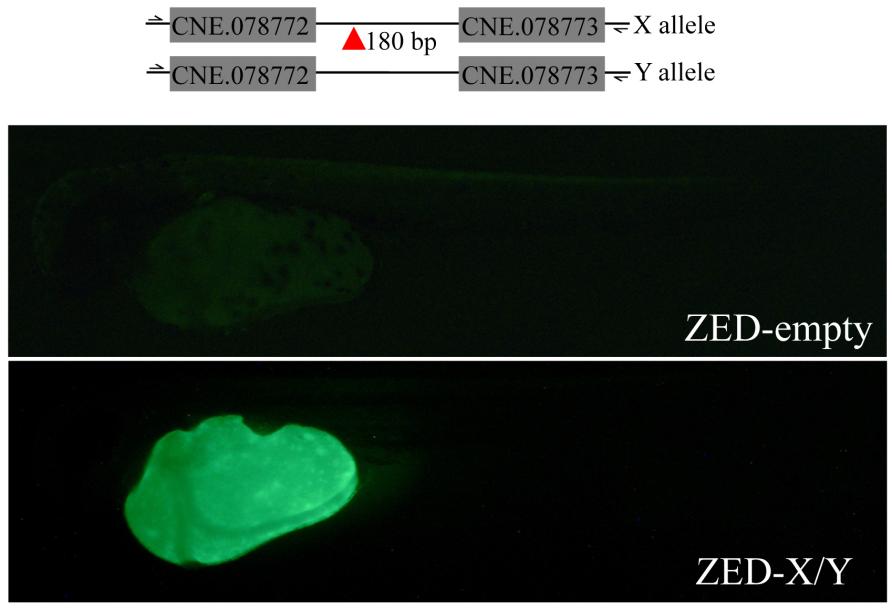


**Fig. S7** Predicted conserved noncoding elements flanking transposon *drbx1* enhance reporter GFP expression. Both X allele and Y allele, including both CNE.078772 and CNE.078773 while the X allele has a transposon *drbx1* insertion between the two CNEs, were constructed into zebrafish enhancer detection vector (ZED). Both X and Y alleles enhance reporter GFP expression in the yolk of fighting fish embryos at 48 hpf, in comparison to the ZED empty vector.


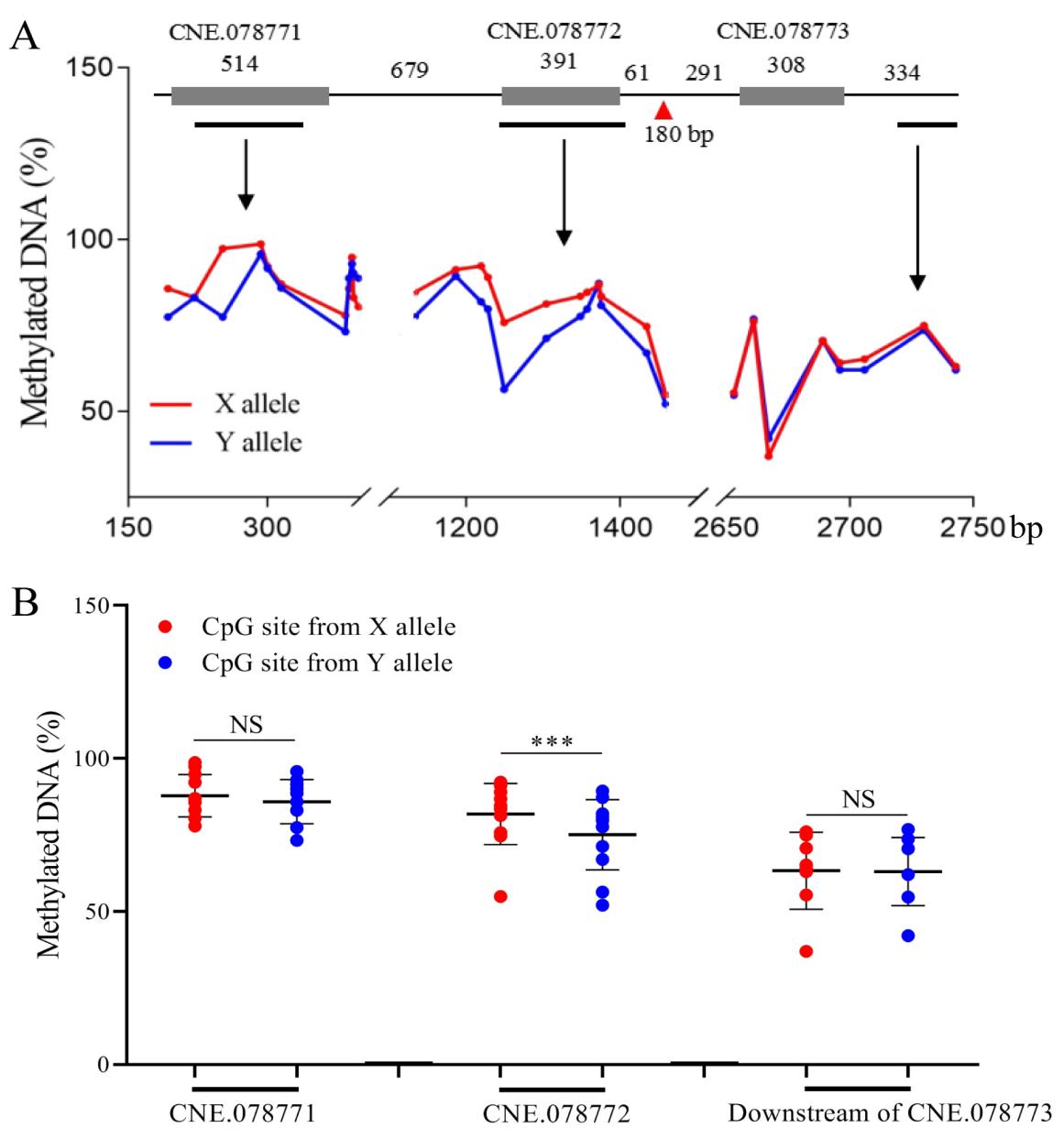


**Fig. S8** Difference in methylation profile in the genomic regions flanking the transposon *drbx1* insertion site between X and Y alleles. A, difference in proportion of methylated CG sites (%) in the genomic regions flanking the transposon *drbx1* insertion site between X and Y alleles, revealed by sequencing PCR products that were produced by using templates of bisulfite-converted whole genomic DNA. The DNA was isolated from trunks of embryos at 3 dpf, separately for XX and YY genotypes. Each dot indicates one CpG site. Predicted conserved noncoding elements (CNEs) and PCR targets for bisulfite sequencing are indicated above. There is no CG site in CNE.078773. B, statistical significance of the differences in methylation profile throughout all CpG sites between X and Y alleles separately for the above three genomic fragments, examined by paired *t*-test, where mean± SD is shown, and NS and *** indicate not significant and *P* < 0.001, respectively.


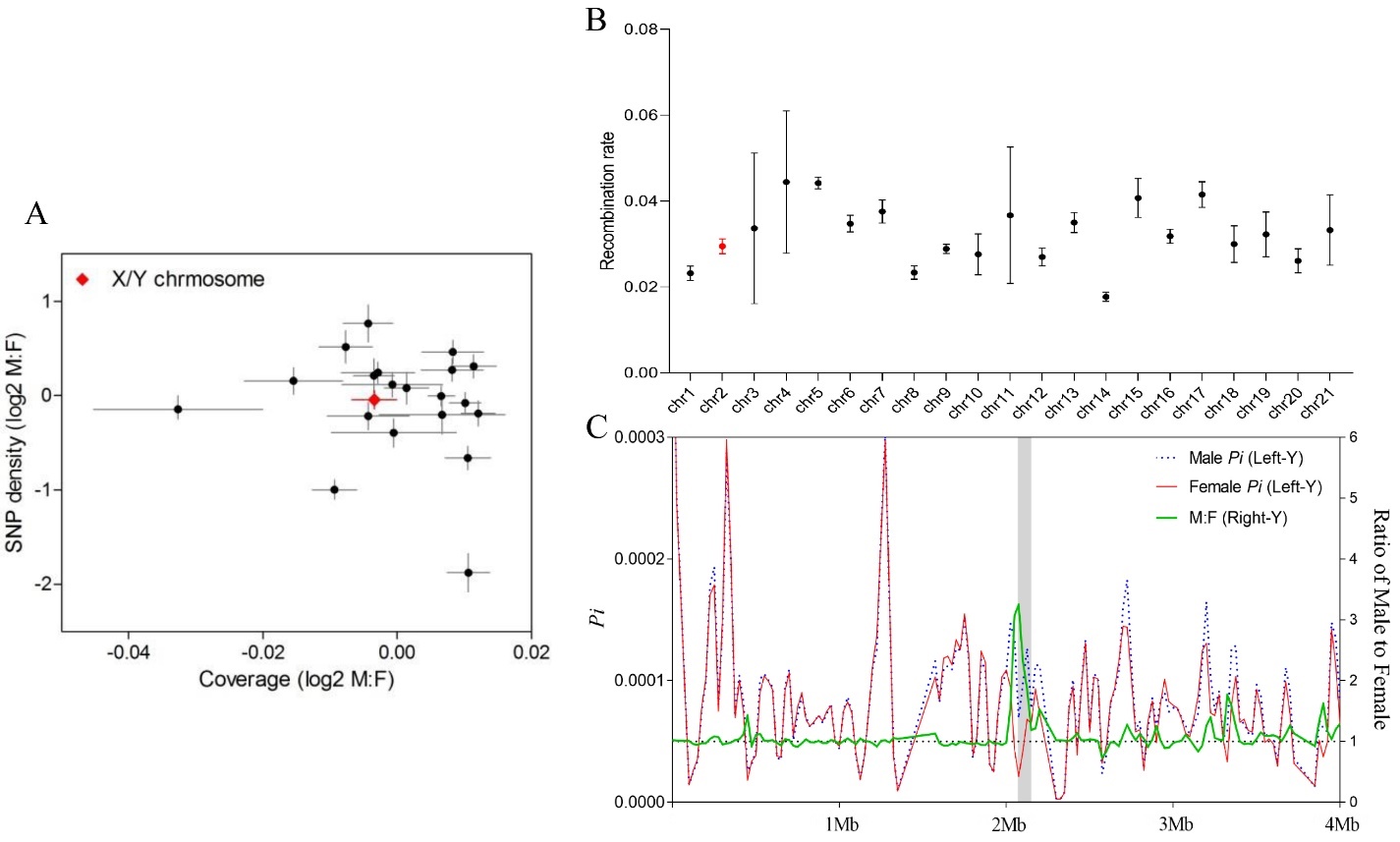


**Fig. S9** Restricted differentiation between X and Y chromosomes in fighting fish. A, Distribution of SNP density and sequencing reads coverage of XX females in contrast to YY males along X chromosome, in comparison to 20 autosomes. The sex chromosome is plotted in red. Interquartile ranges of both SNP density and reads coverage for each chromosome are plotted along X- and Y-axis, respectively. B, comparison of overall recombination rates between sex chromosome and autosomes. C, Distribution of nucleotide diversity along sex chromosome between males and females, estimated with nonoverlapping 50-kb sliding window. The master SD locus is highlighted in light grey shade.


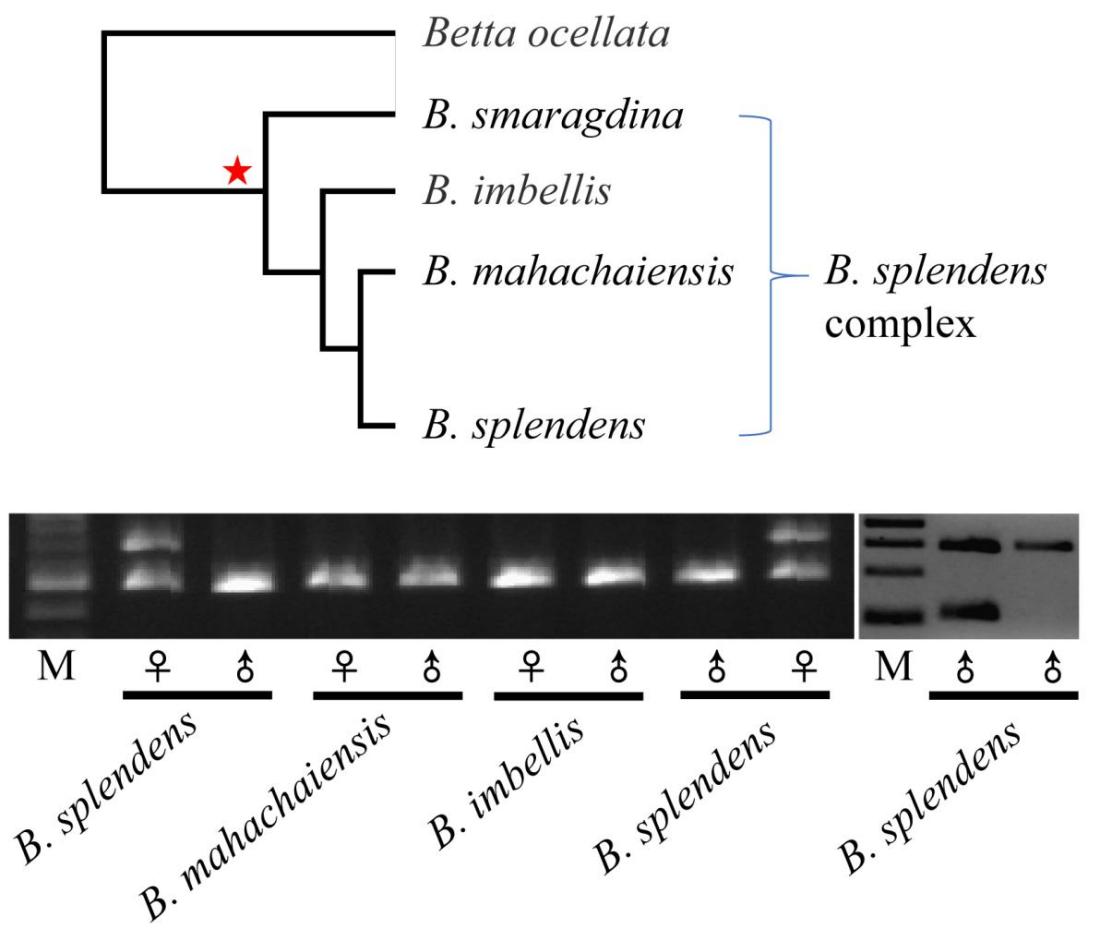


**Fig. S10** Transposon *drbx1* is not associated with phenotypic sex in wild species of *B. splendens* complex. Transposon *drbx1* is absent in both one pair of female and male wild *B. mahachaiensis* and *B. imbellis*. The longer PCR fragments in both gel figures contain the inserted *drbx1*. In six wild *B. splendens*, two females and two males showed XY and YY genotypes, respectively (left gel), while the remaining two males showed XY and XX genotypes, respectively (right gel). This pattern is not agreeing with our observations in domesticated *B. splendens*. It should be noted that the *drbx1* was genotyped using two different primer sets: Sex_GT_F/R and Betta.sex.M180D-1F/R (**supplementary Table S1**) for the left and right gels, respectively. The phylogeny of the *B. splendens* complex is according to a study based on mitochondrial sequences. The red star indicates the *B. splendens* complex.

**Fig. S11** Original uncropped gels for figures and supplementary figures used in this study, where how the gels were cropped is indicated with red box.


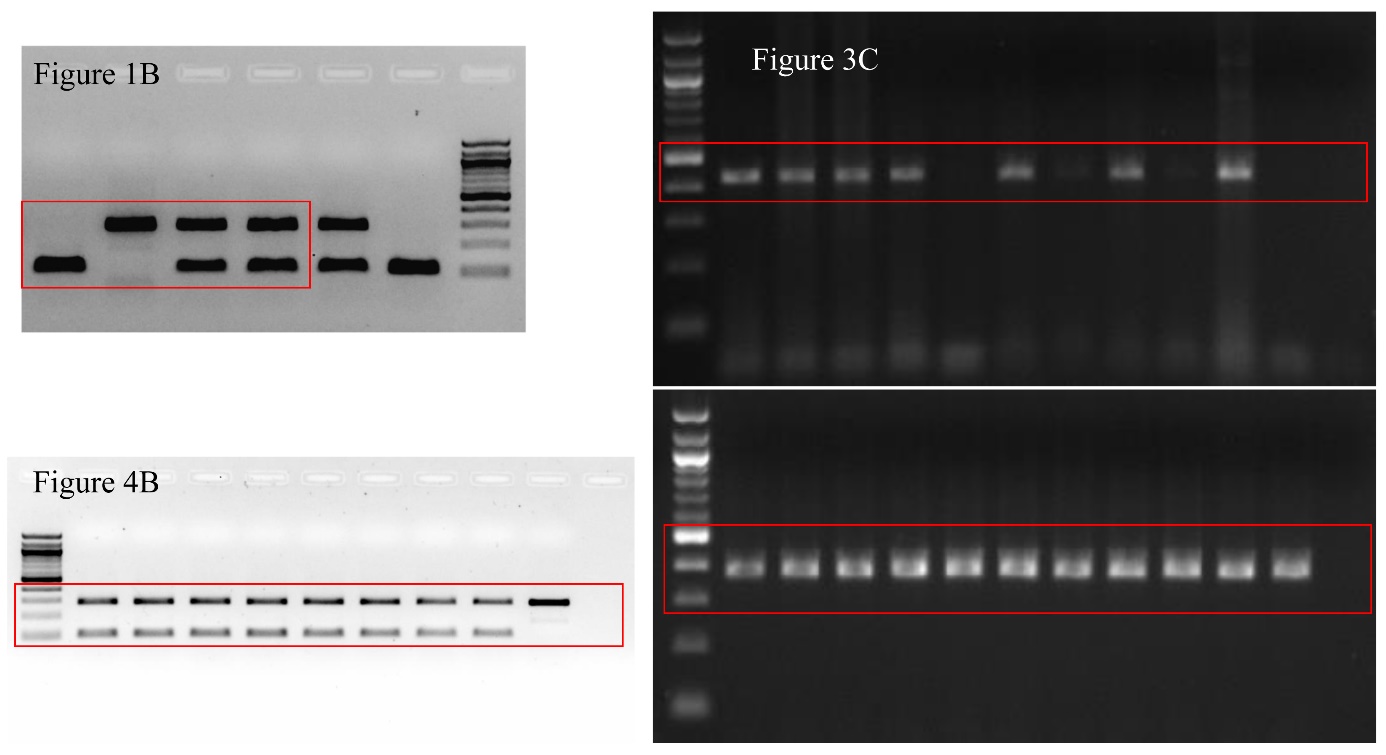


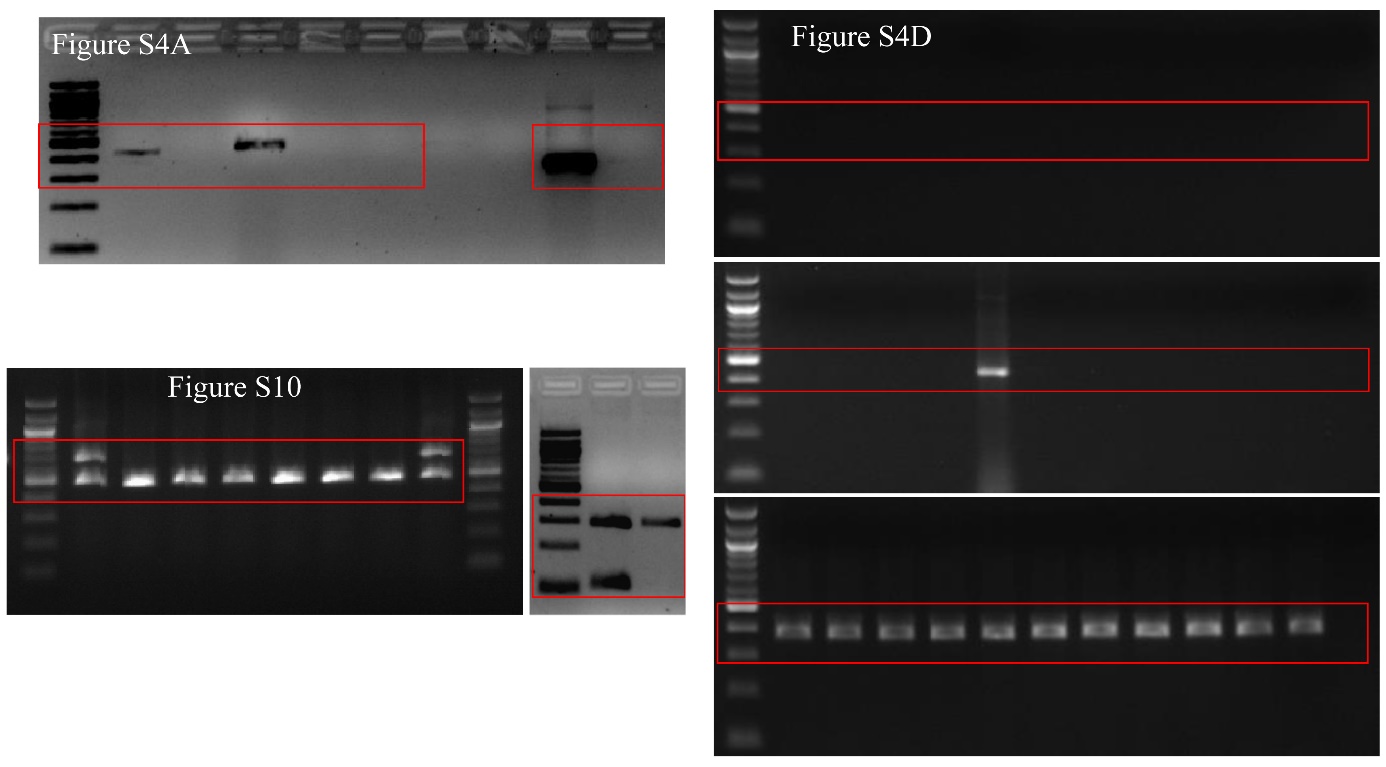


**Table S1** Samples including commercial stocks and wild *Betta* fish used in this study. The number of samples used for GWAS is 509 (indicated with GWAS in the Analysis column), including 502 samples genotyped using RADseq and seven samples resequenced (indicated with resequenced in either Sample/cross or Geographical origin column). A total number of 413 samples were from the three mapping families: P_xx×xy, BM1 and BM2, while the remaining 96 samples were from randomly collected samples (91 individuals) and resequenced samples in China (4 individuals) and Singapore (1 individual with XY genotype) of particular color and fin shape traits.


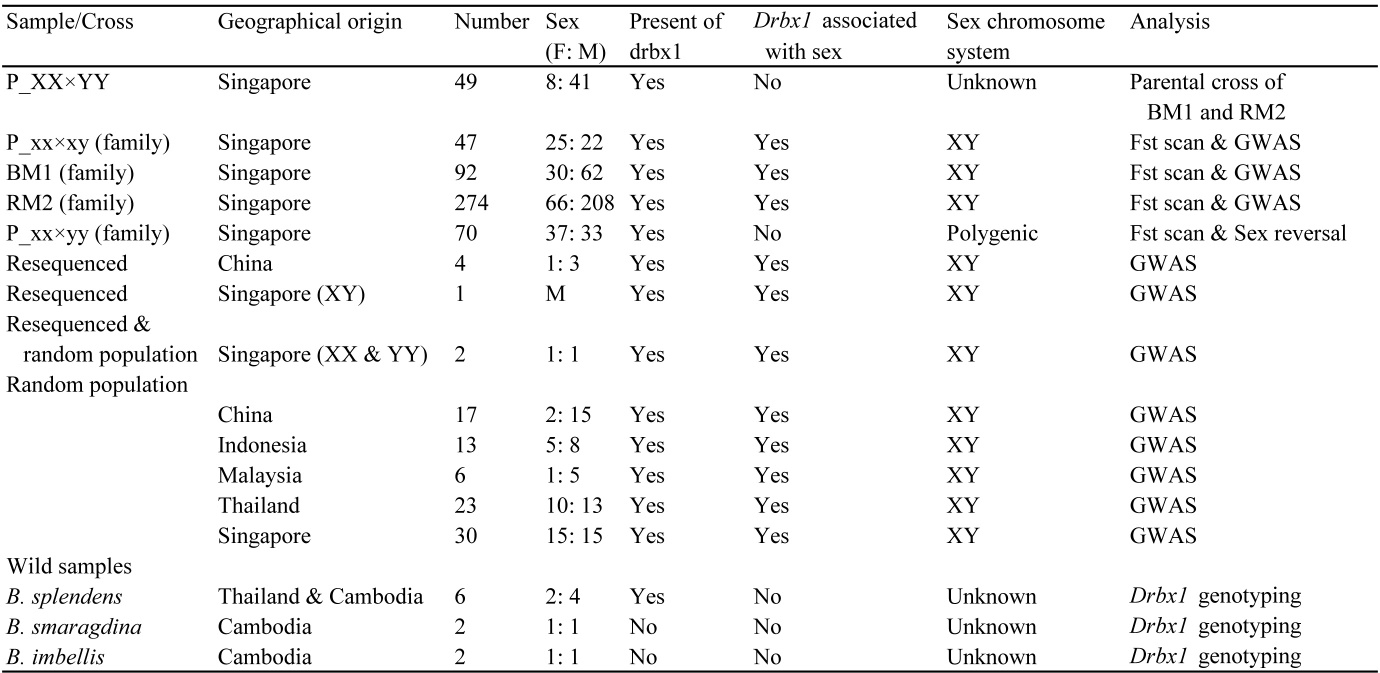


**Table S2** Sequences of primers used in analyzing sex determination in the fighting fish.

| Primer | Sequences (5' to 3') | Purpose/Annotation |
| --- | --- | --- |
| RT_b_actin_F | GGGACGACATGGAGAAGATC | RT PCR for Actin |
| RT_b_actin_R | GCAGTGGTGGTGAAGCTGTA | RT PCR for Actin |
| Betta_ActinF3 | CGGTCGTACCACAGGTATCG | qPCR for Actin |
| Betta_ActinR3 | AGTGGTGGTGAAGCTGTAGC | qPCR for Actin |
| Betta_sex_T1.1F | GCGTCAGTGATGCAGTGTCT | Amplify & sequence SD locus |
| Betta_sex_T1.1R | TTCTCCTTTGAGCGCTGAGG | Amplify & sequence SD locus |
| Betta_sex_T1.2F | CTGAACTGCCAACGCATAGC | Amplify & sequence SD locus |
| Betta_sex_T1.2R | AGTGCTGGTACTCACACTGC | Amplify & sequence SD locus |
| Betta_sex_T1.3F | CCTGAGGAACTGTGGTGTTGT | Amplify & sequence SD locus |
| Betta_sex_T1.3R | GGCCTAAGCAGAGTGTGTGT | Amplify & sequence SD locus |
| Betta_sex_T1.4F | TGTGTGCCACAGCTCTTCAG | Amplify & sequence SD locus |
| Betta_sex_T1.4R | TCACACGCATCCTGGAAACA | Amplify & sequence SD locus |
| Betta_sex_T5.1F | CTGGGGCAGTGGAGACATTA | Amplify & sequence SD locus |
| Betta_sex_T5.1R | TGCAACTGATGTCTGCACCT | Amplify & sequence SD locus |
| Betta_sex_T5.2F | CAGGCACTTGTGGCAGTTTC | Amplify & sequence SD locus |
| Betta_sex_T5.2R | GTACACAAGGGTCATGGGGG | Amplify & sequence SD locus |
| Betta_sex_T5.3F | AACATGATGCAGGTGGCTGT | Amplify & sequence SD locus |
| Betta_sex_T5.3R | ATCACGACACATGCTCCCTG | Amplify & sequence SD locus |
| Betta_sex_T5.4F | GTCAGGTGCTTGACTGCTAA | Amplify & sequence SD locus |
| Betta_sex_T5.4R | AGTGAACTGTGGAACCGCTT | Amplify & sequence SD locus |
| Betta_sex_T1.1-2F | GCGTCAGTGATGCAGTGTCT | Amplify & sequence SD locus |
| Betta_sex_T1.1-2R | ACAGCGTGTGTGTATGTGGT | Amplify & sequence SD locus |
| Betta_sex_T1.2-2F | TGGGGCTTAAGCTGCTGTAC | Amplify & sequence SD locus |
| Betta_sex_T1.2-2R | ACAGGAAGACCAAAGCCATGA | Amplify & sequence SD locus |
| Betta_sex_T1.4-2F | GCCAGTGTACACAGCAACCA | Amplify & sequence SD locus |
| Betta_sex_T1.4-2R | TTCCCCTTCACTCTGTGGGA | Amplify & sequence SD locus |
| Betta_sex_T5.1-2F | TGCCCTCATAGCCTCTGGAT | Amplify & sequence SD locus |
| Betta_sex_T5.1-2R | AGCGTAGGTTGACAGAAGGC | Amplify & sequence SD locus |
| Betta_sex_T5.3-2F | GCTCCTGTAACCTTCAAACAGC | Amplify & sequence SD locus |
| Betta_sex_T5.3-2R | ATCACGACACATGCTCCCTG | Amplify & sequence SD locus |
| Betta_sex_T5.4-2F | TTAACGATTGGGCTGCCAGT | Amplify & sequence SD locus |
| Betta_sex_T5.4-2R | CTCTATGTGGGCGGCGTATA | Amplify & sequence SD locus |
| Dmrt1-FL-1F | CCACGCTTCACTTTTCTTCCTG | Amplify & sequence SD locus |
| Dmrt1-FL-1R | TGCAACAGAGAAATGTGCCTTG | Amplify & sequence SD locus |
| Dmrt3-FL-1F | GCCAACACCTCTTATCCTCCTC | Amplify & sequence SD locus |
| Dmrt3-FL-1R | CTTGACATGCAGCGGTTTTACA | Amplify & sequence SD locus |
| Dmrt2-FL-1F | CAGGCCTCTAACCAGGTTTTGT | Amplify & sequence SD locus |
| Dmrt2-FL-1R | TTTAGTCTGAGCCCAAAGCCAC | Amplify & sequence SD locus |
| Dmrt1-FL-2F | GCGTTAGCTTAGTTAGACGGC | Amplify & sequence SD locus |
| Dmrt1-FL-3F | TGAGTCAAAATGTCTCGATCACA | Amplify & sequence SD locus |
| Dmrt1-FL-2R | TGCAATTGAGGATTAGTTACCTGA | Amplify & sequence SD locus |
| Dmrt1-FL-3R | GCCTTAGAGAAAATAACCTTTAGG | Amplify & sequence SD locus |
| Dmrt3-FL-2F | CAGGTATGAAGCGGCTACGG | Amplify & sequence SD locus |
| Dmrt3-FL-3F | CTCGCTCAGATCTCACCTGC | Amplify & sequence SD locus |
| Dmrt3-FL-4F | AGAGCGTGCTGATCGAAGG | Amplify & sequence SD locus |
| Dmrt3-FL-2R | CAGAGTCAAACACGTGCACG | Amplify & sequence SD locus |
| Dmrt3-FL-3R | CCTGAGCCTCTGCAGGTAAC | Amplify & sequence SD locus |
| Dmrt3-FL-4R | GAGGCGGACTCGGACGTGAA | Amplify & sequence SD locus |
| Dmrt2-FL-2F | ACGCGAATGGTTCCCTGAAA | Amplify & sequence SD locus |
| Dmrt2-FL-3F | GGGCTTGTTTTCTGCTGACC | Amplify & sequence SD locus |
| Dmrt2-FL-2R | AACTGACAGACGGAGCGTTT | Amplify & sequence SD locus |
| Dmrt2-FL-3R | CTGACGTTGGAGGCGATGAG | Amplify & sequence SD locus |
| BSP_F1 | AATTAATTGAGTGAAATTGATAG | Bisulfite sequencing of CNE.078772 |
| BSP_R1 | ACAAATTAAACAAATAACTTCCTAAATATC | Bisulfite sequencing of CNE.078772 |
| BSP_F2 | AGATAATTTGGAAGTATAGATTAAA | Bisulfite sequencing of CNE.078772 |
| BSP_R2 | ACAAACATCAATTACATTTCTCTCA | Bisulfite sequencing of CNE.078772 |
| BSP_F3 | TGTTTTTTATTTTGGTATTTTTTTTA | Bisulfite sequencing of CNE.078771 |
| BSP_R3 | TTACTCCTACATTATTAACCAATCTATCC | Bisulfite sequencing of CNE.078771 |
| BSP_F4 | GGGGTTTTGTTTTATTATAGGGAGT | Bisulfite sequencing of CNE.078773 |
| BSP_R4 | AAACTTCATACTCAACACTCATCCAC | Bisulfite sequencing of CNE.078773 |
| BSP_F5 | TAGTTAGAAGGATTTGGTTTTTTTT | Bisulfite sequencing of CNE.078773 |
| BSP_R5 | AATACTATAATATTTCTCCACACTC | Bisulfite sequencing of CNE.078773 |
| MSP_ctl_F1 | TTGGTTTGATGATGAGGAGT | Bisulfite sequencing control |
| MSP_ctl_R1 | AAACACAAATTAAACAAATAACTTCCTAAA | Bisulfite sequencing control |
| ZED_Dmrt1_F | TTGCAGCGTCCACATCATAG | ZED vector construction |
| ZED_Dmrt1_R | CAAAGAAGACTTTTTCCGTCAG | ZED vector construction |
| pGL3.Prom.dmrt1_F | TTGCAGCGTCCACATCATAG | pGL3 vector construction |
| pGL3.Prom.dmrt1_R | CAAAGAAGACTTTTTCCGTCAG | pGL3 vector construction |
| Sex_GT_F | AGTGGCTTGATCCGACACTC | Genotyping sex at drbx1 locus |
| Sex_GT_R | CCACCTGCATCATGTTTTCA | Genotyping sex at drbx1 locus |
| Betta.sex.M180D-1F | ACAGCACGGAGACATTCAGG | Genotyping sex at drbx1 locus |
| Betta.sex.M180D-1R | ACAGCCACCTGCATCATGTT | Genotyping sex at drbx1 locus |
| Dmrt1_Q2F | CTGAATGATGCGTCTGATCC | qPCR for Dmrt1 |
| Dmrt1_Q2R | CTGGAGGCTGCACTGATCTA | qPCR for Dmrt1 |
| Dmrt2_QF | GTCATCTTCCCCAACAGCAT | qPCR for Dmrt2 |
| Dmrt2_QR | GGTAGTGGCGGTAGGTGGT | qPCR for Dmrt2 |
| Dmrt3a_Q1F | CCAACGAAAGTCTGGAGAGC | qPCR for Dmrt3 |
| Dmrt3a_Q1R | CTGCTCCTTCTCGCTGGA | qPCR for Dmrt3 |
| Kank1_QF | CACACAAACGCTTCTCCTGA | QPCR for Kank1 |
| Kank1_QR | TTTAGTCGAACCCGATTTGC | QPCR for Kank1 |
| RT_Dmrt1_F | ACTGTCAGTGCGTGAAGTGC | RT-PCR for Dmrt1 |
| RT_Dmrt1_R | GCTGGTCGCTGGGTAGTAAG | RT-PCR for Dmrt1 |
| RT_Dmrt3a_F1 | CGAGAAGTGCATCCTCATCA | RT-PCR for Dmrt3 |
| RT_Dmrt3a_R1 | GTGAGGACTCTCTGCCTTGG | RT-PCR for Dmrt3 |
| RT_Dmrt2_F1 | GCTGGTTGCGAGTTTGAAAT | RT-PCR for Dmrt2 |
| RT_Dmrt2_R1 | GGTTTCCAGCTCCTTGTCAG | RT-PCR for Dmrt2 |
| RT_c9orf117F | GCAGCCATGTCAGAAAAGGT | RT-PCR for C9orf117 |
| RT_c9orf117R | GTCTCTGCTCCTCCTGCATC | RT-PCR for C9orf117 |
| RT_Kank1_F | CTGTGGGCAAGGTGGATACT | RT-PCR for Kank1 |
| RT_Kank1_R | TTGCTGGTGGGGTCTAAAAC | RT-PCR for Kank1 |
| dmrt1_ish_F | ATCTGACGTGCTGCTGGAAA | Primers for in situ hybridization |
| dmrt1_ish_R | GGTGCTGTCACAGTCGATGA | Primers for in situ hybridization |
| e1_gRNA1.1 | GATAGGGGTCCGGTGCTCTC | Sequence for gRNA e1_gRNA1.1 |
| e1_gRNA1.2 | CGCCCAAATGCTCCCGCTGC | Sequence for gRNA e1_gRNA1.2 |
| e2_gRNA2.1 | TTGCTTTGAGAAGGCAGC | Sequence for gRNA e2_gRNA2.1 |
| Dmrt_e1_KOSeqF | ATCGGCCCAGTAACAGACAC | Screen knockout mutant at exon 1 |
| Dmrt_e1_KOSeqR | CACTGACAGTCCCTCCACCT | Screen knockout mutant at exon 1 |
| Dmrt_e2_KOSeqF | AAAGGGGATGTTGTGCTTTG | Screen knockout mutant at exon 2 |
| Dmrt_e2_KOSeqR | TGGGAGATCTTGATAAGGCAAC | Screen knockout mutant at exon 2 |
| mProm_F2 | GCCTGAGCCAATGAAGTGTAG | McrBC assay for promoter |
| mProm_R2 | TATTGTGGTCGGATGCACAC | McrBC assay for promoter |
| mDmrt1_Exon1F | TGAGAAGAAATCCCCACGAG | McrBC assay for Exon1 |
| mDmrt1_Exon1R | GACATGTAGCCGTGGTTCCT | McrBC assay for Exon1 |
| mDmrt1_Exon2F | AAAGGGGATGTTGTGCTTTG | McrBC assay for Exon2 |
| mDmrt1_Exon2R | CTGAAGCAGCAGAGGATGTG | McrBC assay for Exon2 |
| mDmrt1_Exon3F | CCTCCAGTCCGTCATCAAGT | McrBC assay for Exon3 |
| mDmrt1_Exon3R | CACCCACCACTACACCACAG | McrBC assay for Exon3 |
| mDmrt1_Exon4F | TTGCACCAGCATAATTTGGA | McrBC assay for Exon4 |
| mDmrt1_Exon4R | GCTGGTCGCTGGGTAGTAAG | McrBC assay for Exon4 |
| mDmrt1_Exon5F1 | CCTGATTCCGCCTCATCTC | McrBC assay for Exon5 |
| mDmrt1_Exon5R1 | TGTGGAACCGCTTTAGGAAC | McrBC assay for Exon5 |
| m.CNE.078765_F1 | TCACTGCCTCCACTGAACTG | McrBC assay for CNE.078765 |
| m.CNE.078765_R1 | CGTAGCTCCCTCAACCTGAC | McrBC assay for CNE.078765 |
| m.CNE.078766_F1 | CAAGGCAGCTTTGTGTGTGT | McrBC assay for CNE.078766 |
| m.CNE.078766_R1 | ATGCTCAGCACTGGCTTTCT | McrBC assay for CNE.078766 |
| m.CNE.078768_F1 | GTTGCTGTGCTGGCTTTACA | McrBC assay for CNE.078768 |
| m.CNE.078768_R1 | CCCAGAGGACAGAGAACCTG | McrBC assay for CNE.078768 |
| m.CNE.078771_F1 | CTGAACGCCTTGCATAAACA | McrBC assay for CNE.078771 |
| m.CNE.078771_R1 | GCTACTGTGCCCCATCAACT | McrBC assay for CNE.078771 |
| m.CNE.078772F1 | AGTGGCTTGATCCGACACTC | McrBC assay for CNE.078772 |
| m.CNE.078772R1 | TTGGCTTGGGATTGAATAGC | McrBC assay for CNE.078772 |
| m.CNE.078775_F1 | TGAGACCGGATTGTACAGCA | McrBC assay for CNE.078775 |
| m.CNE.078775_R1 | GGCAGCAGCTACAGAAGGAC | McrBC assay for CNE.078775 |
| m_control1F | TGGAAGCAAAAGTTCCCACT | McrBC assay for control |
| m_control1R | CCCATTTGTAGCAGGAGGTC | McrBC assay for control |
